# Structural, Aromatic, Optical and Reactive Features of C_12_-(CO)*_n_* (*n* = 2, 4, 6): A DFT Study

**DOI:** 10.3390/nano16191254

**Published:** 2026-10-03

**Authors:** Xin Chen, Pei-Pei Li, Guang-Nian Xu

**Affiliations:** School of Materials and Chemical Engineering, West Anhui University, Lu’an 237012, China; cx810101@163.com

**Keywords:** C_12_-(CO)*_n_* (*n* = 2, 4, 6), Aromaticity, UV-Vis spectra, adsorption properties, reactive properties

## Abstract

The geometric, electronic, spectroscopic, adsorption, and reactive properties of C_12_-(CO)*_n_* (*n* = 2, 4, 6) were systematically investigated using density functional theory at the ωB97XD/def2-TZVP level. All three molecules adopt planar structures with pronounced bond length alternation along the C_12_ skeleton. Multi-index bonding analyses reveal significant π-electron localization at short C–C bonds rather than full-ring delocalization. As the number of OCCO fusion units increases, molecular symmetry evolves from *C*_2*v*_ to *D*_2*h*_ and further to *D*_3*h*_. Aromaticity assessments using AV1245, HOMA, FLU-π, and ELF-π consistently indicate the absence of global aromaticity; OCCO fusion primarily disrupts π_in_ delocalization while leaving π_out_ conjugation comparatively less affected. Simulated UV-Vis spectra show that increasing OCCO units shifts the dominant absorption band toward the ultraviolet region, with low-energy excitations confirmed as locally excited states dominated by charge redistribution within the C_12_ ring. vdW potential calculations disclose an anomalous adsorption strength sequence for light noble gases (He > Ne), and it is contrary to the counterpoise-corrected interaction energy results. Local electron attachment energy (LEAE) and orbital-weighted dual descriptor (OWDD) jointly demonstrate that the intrinsic electron-accepting reactivity follows C_12_-(CO)_6_ > C_12_-(CO)_4_ > C_12_-(CO)_2_, qualitatively consistent with LUMO energy lowering yet modulated by electron-attachment relaxation effects. This work provides theoretical insights into the structure–bonding–reactivity relationships of OCCO-fused carbon macrocyclic systems.

## 1. Introduction

Cyclo[n]carbons are a family of monocyclic ring compounds which are composed entirely of sp-hybridized carbon atoms; they have attracted considerable attention in recent years. Among this family, cyclo[18]carbon (C_18_) has been the subject of extensive theoretical [1,2,3,4,5,6,7,8] and experimental investigations since its first condensed-phase observation [9,10]. More recently, the on-surface method has been successfully applied to the syntheses of cyclo[n]carbon (*n* = 10, 12, 14, 20) [11,12], marking a significant advance in the experimental accessibility of smaller cyclocarbons. Acetylene pyrolysis in a neon atmosphere under pressure may provide another method for synthesizing the C_12_ ring [13].

Theoretical studies have revealed that C_12_ exhibits pronounced anti-aromatic character, with in-plane and out-of-plane double π anti-aromaticity [14,15]. Recently, the potential applications of cyclo[n]carbon systems have attracted growing theoretical interest. Screening of M_3_O@C_2*n*_ (*n* = 5–10, M = Li, Na, and K) confirmed that Li_3_O@C_20_ exhibits the best nonlinear optical (NLO) properties among all the involved species [16]. Anti-aromatic cyclo[4n]carbon-based frameworks (*n* = 3–8) may show nonlinear transport behavior when coupled with different electrodes [17]. Calculations on gas storage and sensing of cyclo[2*n*]carbon (*n* = 6–8) indicate that the adsorption strength for gases increases with the value of *n* and the polarity of gas molecules. Meanwhile, the adsorption stability is mainly due to London dispersion forces [18].

In contrast to cyclo[18]carbon (C_18_), investigations into cyclo[12]carbon (C_12_) are far less in-depth. C_18_-(CO)_6_ (the fusion of three ethylenedione OCCO units with the C_18_ ring) has been used as a precursor for synthesizing C_18_ [9]. But up to now, the precursors for synthesizing C_12_ are limited to halogenated polycyclic aromatic hydrocarbons (PAHs) and acetylene [11,13]. It remains an open question whether C_12_-(CO)*_n_* (*n* = 2, 4, 6) could serve as potential precursors for preparing C_12_. On the other hand, geometries, aromaticity and nonlinear properties of C_18_-(CO)*_n_* (*n* = 2, 4, 6) have been comprehensively investigated. However, because the C_12_ and C_18_ rings have drastically different aromaticity, namely, doubly antiaromaticity [11,12] vs. doubly aromaticity [1,19], it is unclear whether the fusion of OCCO onto C_12_ and C_18_ rings induces identical effects. All these aspects are not well understood.

Inspired by these considerations, we designed C_12_-(CO)*_n_* (*n* = 2, 4, 6), in which the C_12_ ring is fused with varying numbers of ethylenedione (OCCO) four-membered fragments. This series provides a suitable platform to investigate how the number of -CO groups modulates the geometric, electronic, spectroscopic, and reactive properties of the C_12_-based macrocyclic framework. Through comprehensive density functional theory (DFT) calculations at the ωB97XD/def2-TZVP level, we have characterized these systems from multiple perspectives: geometric structures and bonding characteristics (bond lengths, bond orders), π-electron distribution (IRI-π, LOL-π), orbital interactions and charge transfer (EDD, ECDA), aromaticity (AV1245, HOMA, FLU-π, ELF-π), UV-Vis absorption spectra (including hole–electron analysis), van der Waals and electrostatic potentials, and local reactivity (LEAE and OWDD).

The present work intends to study the relationship between structure, properties and reactivity for C_12_-(CO)*_n_* macrocycles, revealing how incremental carbonyl fusion progressively tunes the electronic structure and enhances the intrinsic electron-accepting reactivity. These findings may offer theoretical guidance for the rational design and functional modification of related carbon-oxide cyclic π-systems.

## 2. Computational Details

Geometry optimizations and frequency calculations (at the ωB97XD [20]/def2-TZVP [21] level) and UV-Vis spectra calculations (at the TD-ωB97XD/def2-TZVP level) were performed using Gaussian 16 Revision C.02 software [22]. All other calculations were carried out using Multiwfn 3.8 (dev) [23,24]. Except for ESP (electrostatic potential) and LEAE isosurfaces generated by VMD [25], all other graphics were produced with Multiwfn 3.8 (dev) [23,24]. The use of ωB97XD/def2-TZVP was based on many previous similar studies [2,3,5,8,26,27,28,29,30]. Tight self-consistent field (SCF) convergence criteria were used for structural optimization and TD-DFT calculations, with thresholds of 1.00 × 10^−8^ for the root mean square (RMS) variation in the density matrix, 1.00 × 10^−6^ for its maximum change, and 1.00 × 10^−6^ for the energy change.

## 3. Results

### 3.1. Geometric Structures

**Figure 1:**
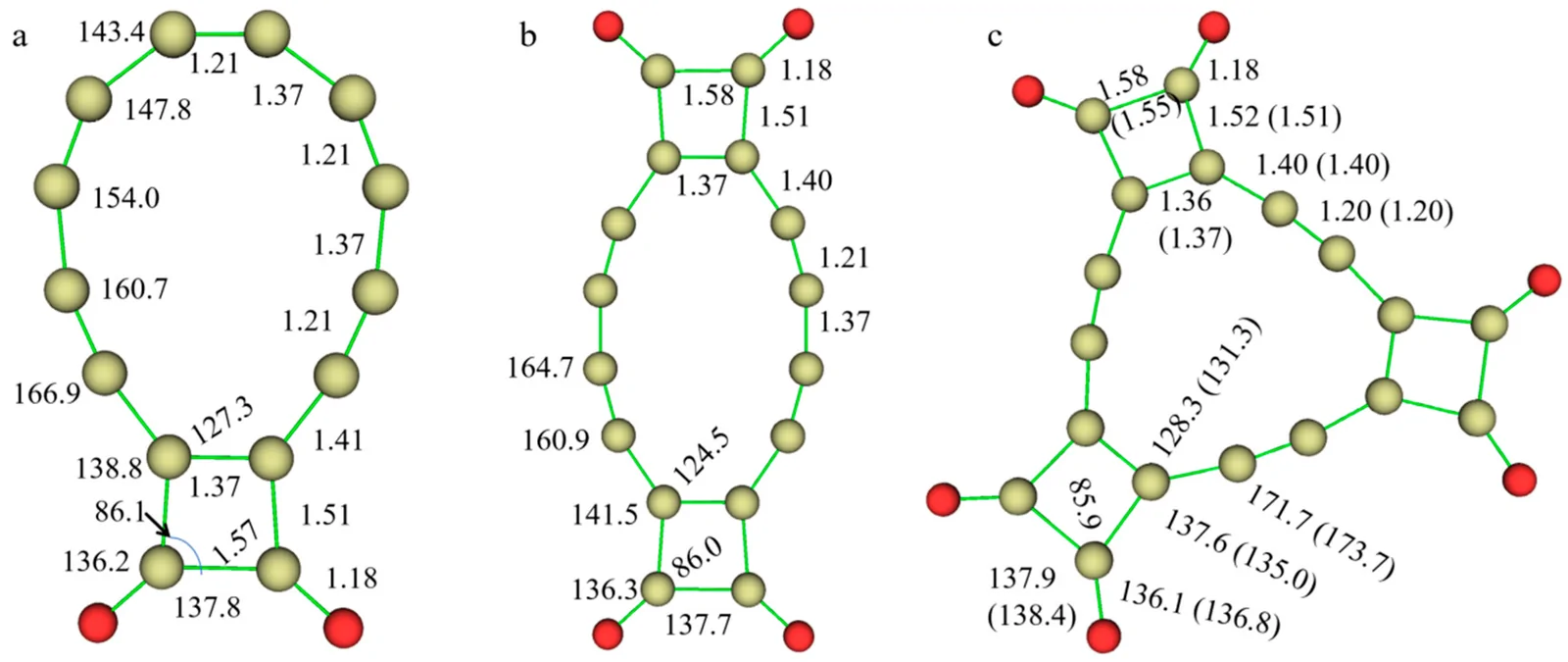
Optimized structural parameters of C_12_-(CO)*_n_* (*n* = 2 (**a**), 4 (**b**) and 6 (**c**)) (data in parentheses are crystal structure data of C_18_-(CO)_6_). Bond angles (left) are given in ° and bond lengths (right) are given in Å. C and O atoms are in orange and red, respectively.

The optimized Cartesian coordinates for C_12_-(CO)*_n_* (*n* = 2, 4, and 6) at the ωB97XD/def2-TZVP level are listed in Table S1. All three compounds are planar, with MPP (molecular planarity parameters) and SDP (deviation from plane) [31] of 0 Å. C_12_-(CO)*_n_* can be regarded as a distorted closed-shell ethylenedione molecule (OCCO) bonded to the C_12_ ring [32,33]. As shown in Figure 1, all the C–C bonds can be divided into five groups: C–C bonds of the C_12_ ring (the shortest 1.20–1.21 Å, intermediate 1.36–1.37 Å, and the longest 1.40–1.41 Å), C–C bonds of OCCO moieties (1.57–1.58 Å), and C–C bonds linking the C_12_ ring and OCCO (1.51–1.52 Å). For each classified bond type, the corresponding bond lengths vary by less than 0.01 Å across the three systems, indicating negligible bond length discrepancy. In contrast, distinct bond angle differences are observed. The internal angle of the four-membered ring (4MR) varies slightly across the three systems. The most prominent structural difference lies in the bond angles of the C_12_ ring backbone and the junction between the C_12_ ring and the 4-membered ring. For the latter, the outer angle changes from 138.8° (*n* = 2), to 141.5° (*n* = 4), and 137.6° (*n* = 6), while the corresponding inner angle varies from 127.3° (*n* = 2), to 124.5° (*n* = 4), and 128.3° (*n* = 6). Such systematic angular rearrangement is induced by directional stretching of the C_12_ ring exerted by OCCO moieties at different positions. These bond length and bond angle values are nearly identical to those in the crystal structure data of C_18_-(CO)_6_ [33].

OCCO can significantly distort the shape of the C_12_ ring. The point groups for C_12_-(CO)*_n_* (*n* = 2, 4, and 6) are *C*_2*v*_, *D*_2*h*_, and *D*_3*h*_, respectively. And the respective cavity diameters of the C_12_ ring are 1.006 Å, 0.445 Å, and 1.042 Å (Figure S1). The cavity diameter of the C_12_ ring for C_12_-(CO)_4_ is far smaller than those of C_12_-(CO)_2_ and C_12_-(CO)_6_; this is because of the strong stretching effect from the two opposite terminal OCCO groups. This discrepancy in cavity diameter can significantly modulate their van der Waals (vdW) adsorption and steric effects.

To evaluate the bond orders of C–C bonds and C–O bonds, five types of bond orders (Mayer [34], Wiberg [35], Laplacian [36], Mulliken [37] and Fuzzy [38]) were calculated (Figure S2 and Table S2). Mulliken bond order (MBO) is excluded from further analysis, because its arbitrary equal partitioning of overlap cross-terms leads to quantitative and qualitative errors for chemical bonds in C_12_-(CO)*_n_* [37].

Mayer, Wiberg, Fuzzy and Laplacian bond orders show consistent trends that correlate well with computed bond lengths. Distinct bond-order alternation is observed along the C_12_ backbone for all three species. High-order C–C bonds (Mayer > 2.5, Wiberg > 2.4) feature prominent multiple-bond character; they originate from local π-electron accumulation, and correspond to short bond lengths near 1.21 Å. The adjacent linkages exhibit intermediate bond-order values (1.1–1.5) and intermediate bond lengths (~1.37 Å). This alternation reveals π-electrons are localized more on high-order sites instead of uniformly delocalized over the whole ring.

Within the four-membered cycle of all three species, the Mayer, Wiberg, Fuzzy and Laplacian bond orders of the C–C bonds in OCCO moieties and the C–C bonds between OCCO and C_12_ ring are all slightly lower than 1.0. It indicates that these C–C bonds are all single bond. But for the fused C–C bonds between the four-membered cycle and the C_12_ ring, the four kinds of bond orders are all about 1.5, and slightly larger than those of other C–C bonds with the same bond lengths (~1.37 Å). Hence, it can be concluded that the fused C–C bond has more π-electrons delocalized. Mayer, Wiberg and Fuzzy bond orders of C–O bonds are all in the range of 2–3; it implies that C–O bonds lie between those of double and triple bonds. But Laplacian bond orders (LBO) of C–O bonds of all three species are all lower than 2.0, it yields relatively low values for these C–O bonds.

To verify the reliability of structural optimization based on the range-separated ωB97XD functional (22.2% short-range HF exchange and 100% long-range HF exchange, with range separation scale ω = 0.2 bohr^−1^), geometry optimizations were also performed using cam-B3LYP (19% short-range HF exchange and 65% long-range HF exchange, with range separation scale ω = 0.33 bohr^−1^) [39] and pbe1pbe functionals (25% HF exchange at all ranges) [40,41] with the unified def2-TZVP basis set. The maximum structural deviations among the three methods were 0.01 Å for bond lengths and 0.5° for bond angles (Figure S3 and Table S3), demonstrating that all functionals afforded almost consistent geometric configurations for the studied system. A comparison of Mayer bond orders of chemical bonds of C_12_-(CO)*_n_* (*n* = 2, 4, and 6) calculated with the three functionals ωB97XD, CAM-B3LYP, and PBE1PBE using the def2-TZVP basis set was also performed (Table S4). The three methods yield consistent trends for the variation in Mayer bond order. (1) The C–C bond orders on the C_12_ ring exhibit an uneven bond order alternation. (2) All C–O bond orders are slightly greater than 2. (3) The C–C bonds within the OCCO moiety and the C–C bonds linking OCCO to the C_12_ ring are lower than 1.0. Hence, the ωB97XD/def2-TZVP level is reliable for analyzing the structures and chemical bonds of these compounds. Unless otherwise specified, the structures optimized at the ωB97XD/def2-TZVP level were adopted for all subsequent calculations.

### 3.2. Character of Bonding

**Figure 2:**
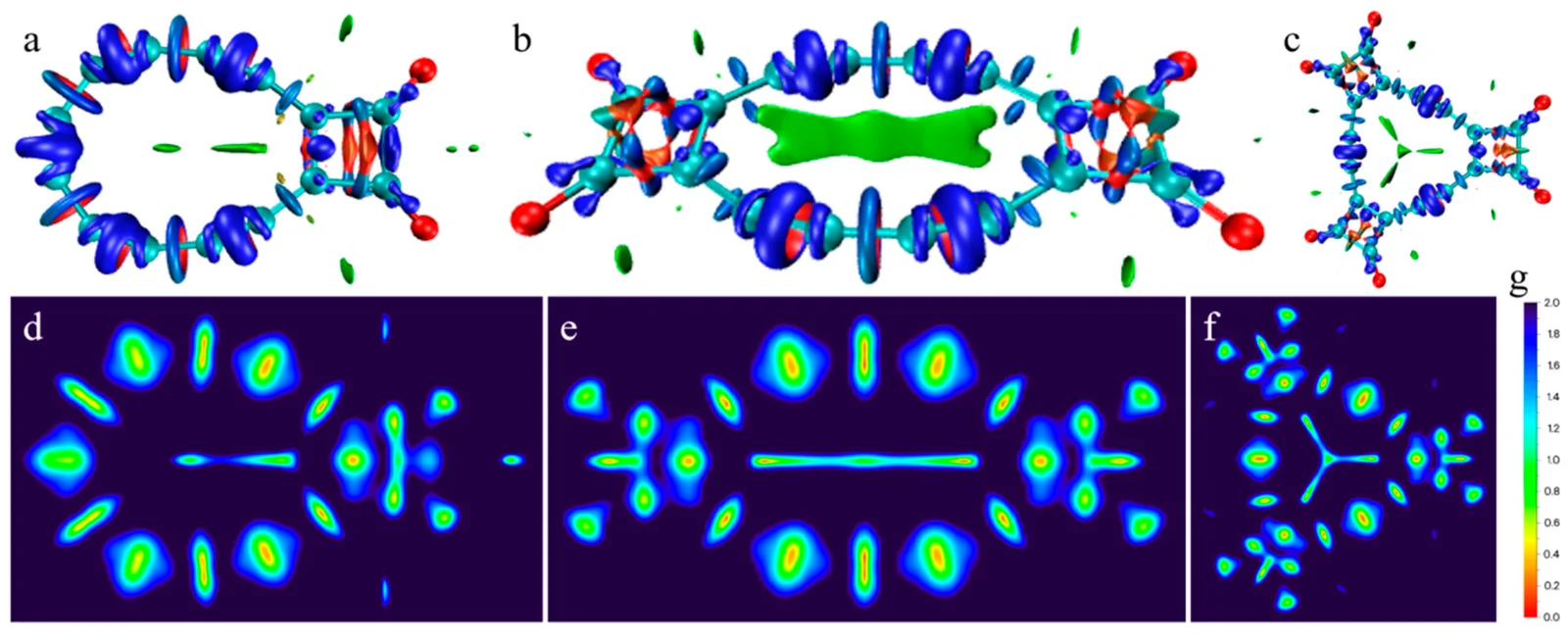
IRI-π isosurfaces (the first row, isovalue = 1.2 a.u.) and IRI-π color-filled maps (the second row) at 0.5 Å above the ring plane of C_12_-(CO)*_n_* ((**a**,**d**) for *n* = 2, (**b**,**e**) for *n* = 4, (**c**,**f**) for *n* = 6). The scale of color bar for IRI-π color-filled maps (**g**) is given in a.u.

To further visualize the π-electron distribution and intramolecular π interactions and verify the above bonding characteristics, IRI-π analyses were conducted for all three C_12_-(CO)*_n_* systems (Figure 2). For the three kinds of C-C bonds of the C_12_ ring (the shortest C–C bonds of 1.20–1.21 Å, intermediate C–C bonds of 1.36–1.37 Å, and the longest C–C bonds of 1.40–1.41 Å), there are only annular IRI-π isosurfaces around the two former C–C bonds. And IRI-π isosurfaces of the shortest C–C bonds are broader than those of intermediate C-C bonds of 1.36–1.37 Å, implying that the shortest C–C bonds have larger π electron density. For the longest C–C bonds of 1.40–1.41 Å, the absence of annular IRI-π isosurfaces predict the weakest π electron density. The IRI-π color-filled maps can provide the same perspective as IRI-π isosurfaces. The blue IRI-π isosurfaces in the cavity of the C_12_ ring indicate the vdW interaction (Figure S4), and the fatter blue IRI-π isosurfaces within the C_12_ ring of C_12_-(CO)_4_ than in the other two species reveal the stronger vdW interaction. This is consistent with its smallest cavity diameter. The red IRI-π isosurfaces in the four-membered cycle demonstrate the notable repulsion in the four-membered cycle. In general, IRI-π isosurfaces imply that π-electrons are delocalized mainly at the shortest and the intermediate C–C bonds of the C_12_ ring, demonstrating the localized nature of π-electron distribution within the C_12_ skeleton.

The IRI-π color-filled maps can provide the same perspective as IRI-π isosurfaces. The simulated STM (Scanning Tunneling Microscopy, Figure S5) image of C_12_-(CO)*_n_* (*n* = 2, 4, and 6) also confirms the electron density difference as IRI-π isosurface.

**Figure 3:**
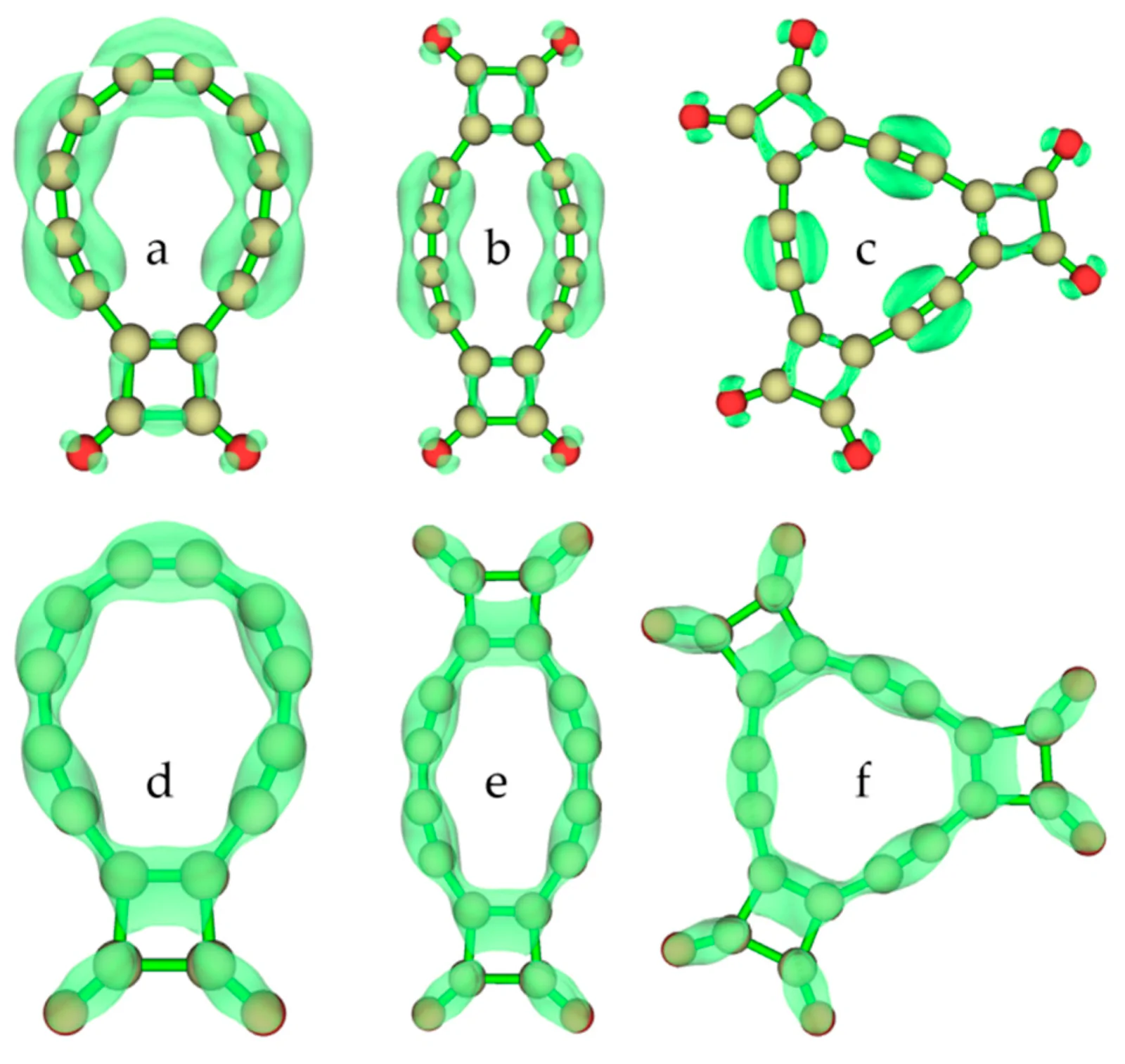
Isosurfaces of LOL-π_in_ (the first row) and LOL-π_out_ (the second row) of C_12_-(CO)*_n_* (*n* = 2 (**a**,**d**), 4 (**b**,**e**), and 6(**c**,**f**)). (Isovalue = 0.4 a.u.).

The π delocalization character can be intuitively reflected by localized orbital locator for π electrons (LOL-π) [42]. As seen in Figure 3, isosurfaces of LOL-π_in_ and LOL-π_out_ of C_12_-(CO)*_n_* (*n* = 2, 4, and 6) show the following characteristics: (1) -CO groups can exert a more pronounced effect on LOL-π_in_ than on LOL-π_out_; local delocalization is more obvious for LOL-π_in_ than LOL-π_out_, and this effect increases with the number of -CO groups. (2) Whether for LOL-π_in_ or LOL-π_out_, the C–C bonds of 1.40–1.41 Å bears relative smaller π electrons compared to other C–C bonds of the C_12_ ring, as can be seen in the truncated positions of LOL-π_in_ and LOL-π_out_ isosurfaces. (3) The electron localization of π electrons on the fused C–C bonds and the C–O bonds of -CO groups mainly arises from π_out_ electrons.

### 3.3. Orbital Interaction Analyses

When OCCO are bonded to the C_12_ ring, electron density difference (EDD) calculations were performed for C_12_-(CO)*_n_* (*n* = 2, 4, 6). The calculation results confirm that electron density changes mainly occur on the OCCO and the C–C bonds of 4MR (Figure S6). Extended charge decomposition analyses (ECDA) [43,44] results indicate that for C_12_-(CO)*_n_* (*n* = 2, 4, 6), the net numbers of electrons transferred from the C_12_ ring to OCCO are 0.1004 e, 0.1395 e, and 0.2258 e, respectively. In other words, each -CO group obtains 0.0502 e, 0.0349 e, and 0.0376 e from the C_12_ ring in C_12_-(CO)*_n_* (*n* = 2, 4, 6), respectively.

Orbital interaction diagrams of C_12_-(CO)*_n_* (*n* = 2, 4, 6), and contributions of fragment (C_12_ ring and closed-shell OCCO) orbitals to their frontier molecular orbitals are shown in Figure S7 and Table S5. The LUMO (the lowest unoccupied molecular orbital) of C_12_-(CO)_6_ is composed of C_12_ π_out_ orbital (89.97%) and OCCO σ orbital (8.21%), while its HOMO (the highest occupied orbital) consists of C_12_ π_in_ orbital (38.27%), OCCO π_in_ orbital (25.22%) and OCCO σ orbital (31.22%). These compositional differences result in the different bonding characters of the longer C–C bonds of the C_12_ ring of its LUMO and HOMO. The former bears more π- bond character, and the latter has only σ- bond character. Orbital interaction analyses are helpful for understanding bonding character, π electrons delocalization and localization scenario. The combination of OCCO with the C_12_ ring will lead to the dramatic C–C–C angle distortions (124.5–128.3°, Figure 1) in C_12_ ring near OCCO, and obviously decrease π_in_ electron delocalization in this region. Hence, the junction C–C bonds between the C_12_ ring and OCCO moieties have no annular IRI-π isosurfaces, and no LOL-π_in_ delocalization.

### 3.4. Aromaticity Analyses

The aromaticity and π-conjugation characteristics of the C_12_ ring of C_12_-(CO)*_n_* (*n* = 2, 4, and 6) were systematically analyzed using geometric (HOMA, Harmonic Oscillator Model of Aromaticity [45]), real-space topological (ELF-π, Electron Localization Function-π [46]), and orbital/electron-delocalization (AV1245 [47] and FLU-π, Aromatic Fluctuation Index [48]) descriptors to reveal their intrinsic conjugation features. The physical meaning and limitations of HOMA, FLU-π, ELF-π and AV1245, together with the rationale for their combined application, are given in Text S1. The π_in_ and π_out_ molecular orbitals of C_12_-(CO)*_n_* (*n* = 2, 4, and 6) are shown in Figure S8. Unless otherwise specified, the subsequent aromaticity discussion is restricted to the C_12_ ring of C_12_-(CO)*_n_* (*n* = 2, 4, 6). Physical meaning, thresholds, and limitations of the aromaticity descriptors are listed in Text S1 [45,47,48,49,50,51,52,53,54,55].

The larger the AV1245 values, the stronger the delocalization and the higher the aromaticity. For C_12_-(CO)*_n_* (*n* = 2, 4, 6), the total AV1245-π decreases monotonically from 1.04 (*n* = 2) to 0.56 (*n* = 4) and further to 0.40 (*n* = 6), indicating a progressive attenuation of π-aromatic character upon increasing -CO substitution (Table 1). An interesting inversion between AV1245-π_in_ and AV1245-π_out_ is observed along this series. At *n* = 2, AV1245-π_in_ (0.60) exceeds AV1245-π_out_ (0.44). As the number of -CO groups increases to *n* = 4 and *n* = 6, AV1245-π_in_ drops sharply to 0.14 and 0.09, respectively, becoming substantially smaller than the corresponding AV1245-π_out_ values (0.42 and 0.31). This reversal reveals that -CO groups exert a strong destructive effect predominantly on the π_in_ delocalization of C_12_-(CO)*_n_*, while the π_out_ delocalization is comparatively less perturbed. A similar overall downward trend in AV1245-π is found for the C_18_-(CO)*_n_* (*n* = 2, 4, 6), which falls from 2.69 (*n* = 2) to 2.12 (*n* = 4) and 1.07 (*n* = 6). Distinct from the C_12_ series, AV1245-π_in_ is persistently lower than AV1245-π_out_ across all *n* values for C_18_-(CO)*_n_*. Even though AV1245-π_in_ turns negative (−0.53) at *n* = 6, the AV1245-π_out_ remains considerably high (2.18, 1.89, and 1.60 for *n* = 2, 4, and 6, respectively). Analogous to the C_12_ series, the -CO groups influence π_in_ delocalization more significantly than π_out_ delocalization for C_18_-(CO)*_n_* [5].

The HOMA values for C_12_-(CO)*_n_* are −2.41, −1.87, and −1.26 for *n* = 2, 4, and 6, respectively. All values are negative, and their gradual increase toward zero suggests a modest geometric regularization with increasing CO substitution. Concurrently, the AV1245-π_out_ value decreases continuously from 0.44 to 0.31, while FLU-π increases from 0.498 to 0.773, indicative of deteriorated multicenter conjugation and growing π-electron inhomogeneity. C_12_-(CO)_4_ exhibits the lowest AV_min_-π_out_ (0.107), indicating a pronounced local disruption of cyclic conjugation at specific sites. The higher-symmetry C_12_-(CO)_6_ shows a slightly higher AV_min_-π_out_ (0.153) and a more uniform π distribution, yet retains the weakest overall multicenter delocalization within the series.

The real-space ELF-π_out_ results exhibit a slight trend opposite to that of the orbital descriptor AV1245-π_out_ (Figure S9, Table 1 and Table 2). The ELF-π_out_ bifurcation values marginally increase from 0.415 to 0.425 with increasing -CO substitution (Table 2). This difference arises mainly from the different ways in which the two descriptors are constructed. Some selected ELF-π_in_/ELF-π_out_ orbitals are not strictly confined to the C_12_ ring; they may include contributions from the π orbitals and even partial σ orbitals of the 4MRs (Figure S9). In contrast, the evaluation of AV1245-π_in_/AV1245-π_out_, HOMA, and FLU-π involves only the atoms and bonds of the C_12_ ring and therefore does not directly include contributions from the 4MRs. Consequently, ELF-π_out_ reflects the real-space π-basin connectivity that may include contributions from the 4MRs, whereas AV1245-π_out_ provides a more direct measure of the intrinsic π delocalization within the C_12_ ring itself. This difference in construction does not affect the main conclusion, since ELF-π_in_ remains nearly zero (0.0012–0.0065) and far below ELF-π_out_ (0.415–0.425), while AV1245, HOMA, and FLU-π consistently indicate negligible π-aromaticity of the C_12_ ring.

Together with the qualitative LOL-π_in_/LOL-π_out_ analysis (Figure 3), the quantitative AV1245-π_in_/AV1245-π_out_ and ELF-π_in_/ELF-π_out_ indices consistently indicate uneven π_in_ and π_out_ electron delocalization, with the π_in_ delocalization strength gradually decreasing as *n* increases.

In summary, for the C_12_ ring in C_12_-(CO)*_n_* (*n* = 2, 4, and 6), the four descriptors consistently indicate negligible π-aromaticity. HOMA is strongly negative (−2.410, −1.866, and −1.258 for *n* = 2, 4, and 6, respectively), far from the aromatic threshold of 1, indicating pronounced bond-length alternation. FLU-π is large (0.498, 0.652, and 0.773), well above aromatic threshold of 0 and consistent with nonaromatic or antiaromatic character. ELF-π is low (0.187, 0.195, and 0.288), far below the aromatic threshold of 0.70. In particular, ELF-π_in_ is nearly 0 (0.0012–0.0065), whereas ELF-π_out_ is 0.415–0.425, indicating that π localization occurs mainly outside the C_12_ ring rather than along the ring pathway. AV1245 is only 0.40–1.04, much lower than the benzene reference value 10.5–10.7, and AV_min_-π_in_ is only 0.006–0.016, further indicating very weak multicenter π delocalization along the C_12_ ring. Therefore, the present results provide reasonable converging evidence against aromaticity in the C_12_ ring within the limitations of the employed descriptors.

### 3.5. UV-Vis Spectra

**Figure 4:**
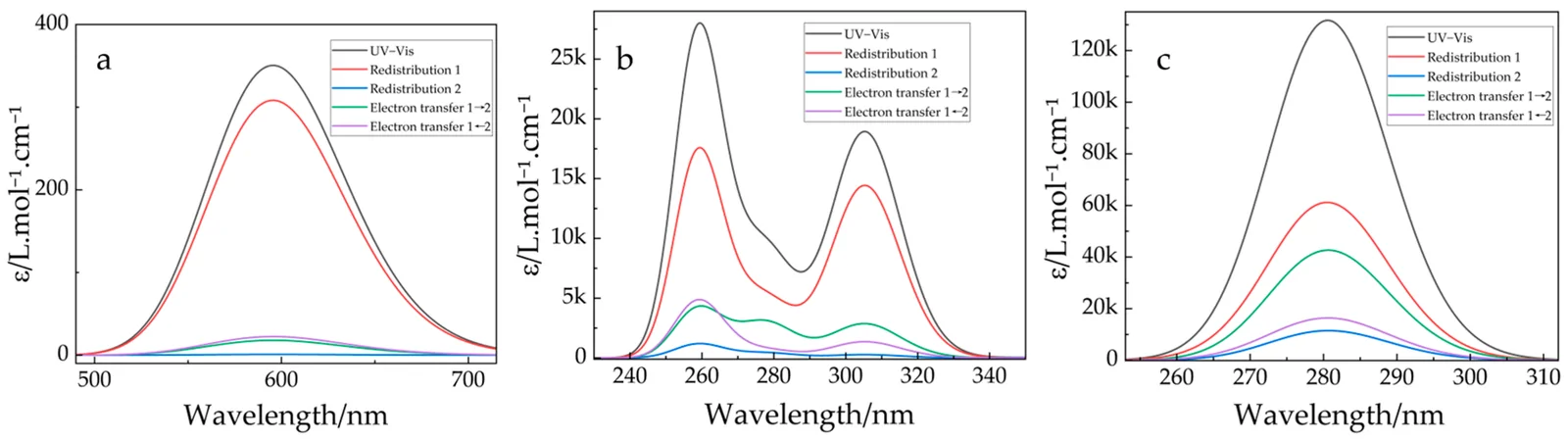
Simulated UV-Vis spectra (black curves) and charge-transfer spectra (CTSs, colored curves) of C_12_–(CO)*_n_* (*n* = 2 (**a**), *n* = 4 (**b**), and *n* = 6 (**c**)) with FWHM 0.3 eV. For (**a**–**c**), fragment 1 and 2 are C_12_ ring and (CO)*_n_*, respectively.

The lowest-energy UV-Vis absorption spectra (oscillator strength *f* > 0.003) [56] and their CTS (charge-transfer spectra) of C_12_-(CO)*_n_* (*n* = 2, 4, and 6) simulated with a full width at half maximum (FWHM) of 0.3 eV are shown in Figure 4. Simulated absorption spectra and CTS of C_12_-(CO)_2_ at 200–715 nm and 200–425 nm are displayed in Figure S10. Main excited-state parameters of the first 20 excited states of C_12_-(CO)*_n_* (*n* = 2, 4, and 6) in the gas phase are listed in Table S6.

Increasing *n* in C_12_-(CO)*_n_* (*n* = 2, 4, and 6) changes the absorption profile from weak transitions to intense UV absorption (Figure 4 and Table S6), and redistributes the oscillator strength from low-lying dark states to higher-energy mixed configurations. For *n* = 2, the absorption band near 595.5 nm is very weak (S2, *f* = 0.0039) and is not the main absorption; the strongest transition lies in the UV region around 260 nm (S11, *f* ≈ 0.07). For *n* = 4 and 6, intense UV bands appear at 305.4 nm (S8, *f* = 0.2100) and 280.7 nm (S11/S12, *f* = 0.7228), respectively, and the maximum oscillator strength increases from 0.07 (*n* = 2) to 0.31 (*n* = 4) and 0.72 (*n* = 6). In contrast, the maximum absorption of C_18_-(CO)*_n_* red-shifts from 225.0 to 294.5 and 344.0 nm [8]. This contrast reflects the distinct electronic response of the doubly anti-aromatic C_12_ ring [11,12] versus the doubly aromatic C_18_ ring [1,2].

To verify the reliability of the UV-Vis spectrum calculations at the TD-ωB97XD/def2-TZVP level, TD-CAM-B3LYP/def2-TZVP calculations were also performed based on the optimized structure at the ωB97XD/def2-TZVP level. As seen in Figure S11, Tables S7 and S8, the differences in the simulated UV-Vis spectra and related excited states parameters (excited energy, excited wavelength, oscillator strength and main molecular orbital transitions) between these two methods are minor. This indicates that the UV-Vis spectrum calculation results at the TD-ωB97XD/def2-TZVP level are reliable. Natural transition orbitals (NTO) analyses (Figure S12) reveal that S8 state of C_12_-(CO)_4_ has mixed characteristics of π_in_→π_in_* and π_out_→π_out_*, while S2 state of C_12_-(CO)_2_ and S11/S12 state of C_12_-(CO)_6_ are almost solely π_out_→π_out_* excitations.

For the overall CTS of both C_12_-(CO)*_n_* and C_18_-(CO)*_n_* (*n* = 2, 4, and 6), the main charge transfer occurs in the following sequence: inside the carbon (C_12_ or C_18_) ring, from the carbon ring to OCCO, from OCCO to the carbon ring, and inside OCCO. The electron redistribution within the carbon ring dominates the electron excitations, and the electron redistribution within the OCCO group is almost negligible [8].

Hole–electron analyses [19] were performed to characterize the excited states responsible for the above-mentioned absorption bands. As shown in Table 3, the relevant states (S2 of C_12_-(CO)_2_, S8 of C_12_-(CO)_4_, and S11/S12 of C_12_-(CO)_6_) all exhibit small *D* values, large Sr values (about 0.8), and negative t values. According to the criteria given in Text S2, these features indicate that the hole and electron are not significantly separated, consistent with locally excited (LE) character. The hole and electron distributions in Figure S13 are mainly located on the C_12_ ring and the –(CO)*_n_* (*n* = 2, 4, and 6) groups and overlap substantially. Consistent with the CTS results (Figure 4), charge redistribution within the C_12_ ring provides the main contribution to these excitations, while the contributions from the OCCO moiety and C_12_ ring **→** OCCO charge transfer differ among the systems.

### 3.6. Analyses of van der Waals (vdW) Potential and Electrostatic Potential (ESP)

In this section, a multi-step computational strategy is used to analyze rare gas adsorption on C_12_-(CO)*_n_*. The UFF (Universal Force Field)-based van der Waals potential (VvdW) [57] delivers preliminary qualitative screening under fixed molecular geometries. SAPT (symmetry-adapted perturbation theory) [58] energy decomposition using Psi4 [59] was performed on geometries optimized at the ωB97XD/def2-TZVP level to compare the relative magnitude of interaction components. BSSE (basis set superposition error)-corrected [60] DFT calculations on fully optimized structures at the ωB97XD/def2-TZVP level were carried out to provide quantitative binding energies and serve as the primary benchmark for adsorption strength.

UFF calculations show that the overall interaction order is Xe > Ar > He > Ne for all C_12_-(CO)*_n_* (*n* = 2, 4, and 6) species (Table S9 and Figure S14). SAPT0 energy decomposition analyses using the SAPT0/jun-cc-pVDZ method indicate that for a given *n* of C_12_-(CO)*_n_*@ He/Ne complexes, all four energy components (electrostatic, exchange repulsion, induction and dispersion) of the C_12_-(CO)*_n_*@Ne exhibit larger magnitudes than those of the C_12_-(CO)*_n_*@He (Table S10).

To verify the reliability of qualitative analysis results of van der Waals potential, the structures of C_12_-(CO)*_n_*@He/Ne (*n* = 2, 4, 6) were optimized at the ωB97XD/def2-TZVP level using Gaussian 16 C.02 to provide geometries for ORCA [61] calculations. ORCA software (version 5.0.4) was utilized to assess basis set convergence and BSSE via multiple computational methods based on the optimized structures at the ωB97XD/def2-TZVP level, owing to its high computational efficiency. Benchmark calculations at the ωB97X-D3BJ level demonstrate that the counterpoise-corrected binding energies ΔE_CP_ of the six C_12_-(CO)*_n_*@He/Ne systems exhibit only minor differences between def2-TZVP and the larger, more polarized def2-QZVP basis set (Table S11). For C_12_-(CO)_2_@He, the ΔE_CP_ value obtained with def2-TZVP also agrees closely with that calculated using the diffuse augmented ma-def2-TZVP basis set. For instance, ΔE_CP_ of C_12_-(CO)*_n_*@He is −0.392 kcal/mol (def2-TZVP), −0.393 kcal/mol (def2-QZVP), and −0.404 kcal/mol (ma-def2-TZVP). These benchmark results validate that the def2-TZVP basis set is adequate for the subsequent data analysis in this work.

Although the UFF-based van der Waals potential correctly captures the qualitative trend of vertical distances between He/Ne and the C_12_ ring, i.e., the vertical distance follows the order (*n* = 4 > *n* = 6 > *n* = 2) for C_12_-(CO)*_n_*@He/Ne (Table S12), it underestimates the absolute values of these vertical distances. As exemplified by C_12_-(CO)_2_@He (2.886 Å, DFT vs. 1.91 Å, UFF) and C_12_-(CO)_2_@Ne (2.889 Å, DFT vs. 2.62 Å, UFF), this underestimation is especially pronounced for He, which may explain the anomalous V_vdW_ trend.

**Figure 5:**
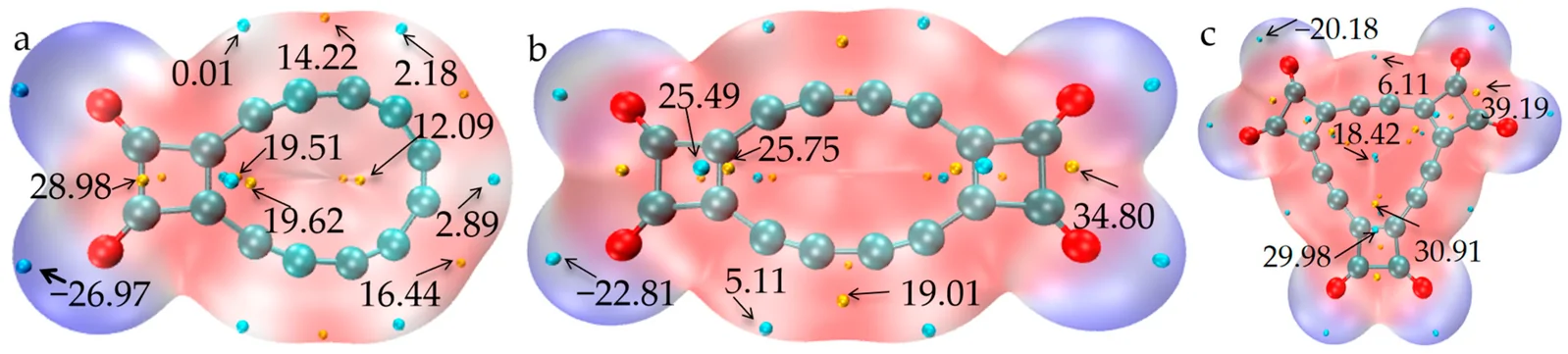
Molecular electrostatic potential extrema of C_12_-(CO)*n*: (**a**) (*n* = 2); (**b**) (*n* = 4); (**c**) (*n* = 6). The marked values correspond to ESP extrema in kcal·mol^−1^. The small yellow spheres and cyan spheres denote local ESP maxima (*V*_S,max_) and local ESP minima (*V*_S,min_), respectively.

Figure 5 further characterizes the electrostatic properties of these planar C_12_-(CO)*_n_* systems by mapping electrostatic potential (ESP) extrema [62,63] on electron-density isosurfaces. Their most negative ESP values are −26.97 (*n* = 2), −22.81 (*n* = 4) and −20.18 kcal·mol^−1^ (*n* = 6), respectively. And these negative ESP extrema are concentrated around oxygen atoms of -CO groups. The most positive ESP extrema of them are 28.98 (*n* = 2), 34.80 (*n* = 4) and 39.19 kcal·mol^−1^ (*n* = 6), respectively. And these positive ESP extrema are all located on both sides of the C–C bonds of OCCO moieties. The second-largest positive ESP extrema are all situated on both sides of the fused C–C bonds between the 4MR and C_12_ ring, and the values are on average 19.56 (*n* = 2), 25.62 (*n* = 4), and 30.45 kcal·mol^−1^ (*n* = 6), respectively. The positions of ESP extrema differ substantially from those of the vdW potential.

### 3.7. Reactivity

**Figure 6:**
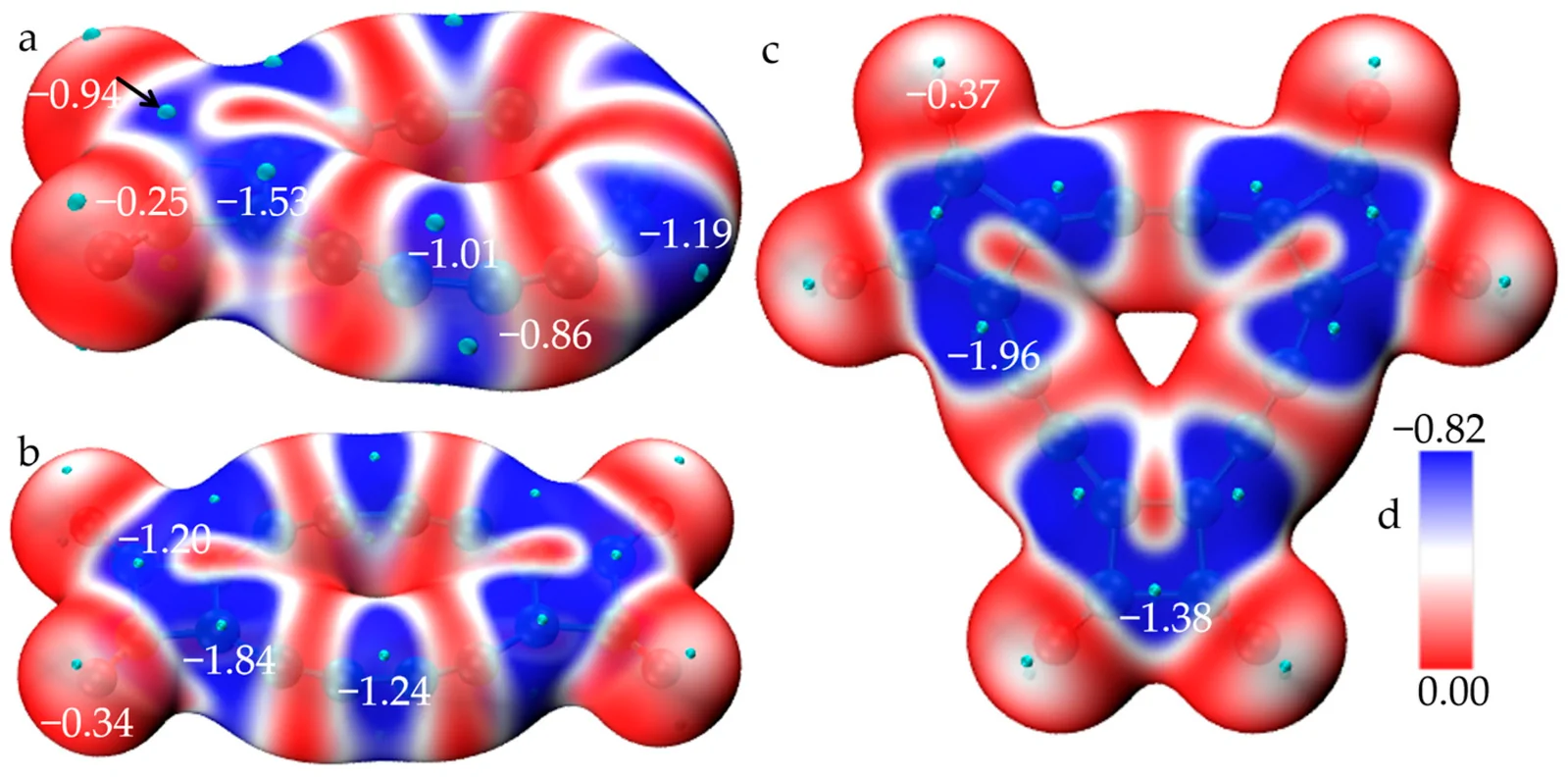
The LEAE (local electron attachment energy) on the molecular surface defined by an electron density of 0.004 a.u. ((**a**): C_12_-(CO)_2_; (**b**): C_12_-(CO)_4_; (**c**): C_12_-(CO)_6_; (**d**): color bar). All values are in eV.

In this section, C_12_-(CO)*_n_* (*n* = 2, 4, 6) are designated as substrates. To explore their reactivity, LEAE [64] and OWDD [65] (Text S3) were combined to characterize the local electronic reactivity (Figure 6 and Figure S15). The dominant nucleophilic sites for all three C_12_-(CO)*_n_* (*n* = 2, 4, 6) are invariably located on both sides of the molecular surface plane of the longest C–C bonds of the C_12_ ring. These sites possess the most negative LEAE values and the most extensive positive OWDD isosurfaces in each system, demonstrating superior electron-accepting affinity and predominant nucleophilic reactivity. Restricted by molecular symmetry, symmetry-equivalent reactive sites exhibit identical electronic characteristics.

The overall nucleophilic susceptibility of the system is gradually enhanced with increasing -CO groups. The most negative LEAE values of the dominant active sites decrease progressively from −1.53 eV for *n* = 2 to −1.84 eV for *n* = 4, and further to −1.96 eV for *n* = 6. This trend is primarily attributed to the cumulative electron-withdrawing effect of -CO groups. Hence, the final reactivity order follows C_12_-(CO)_6_ > C_12_-(CO)_4_ > C_12_-(CO)_2_. As the number of -CO groups increases from *n* = 2 to *n* = 6, both HOMO and LUMO energies shift toward more negative values, accompanied by a gradual reduction in the HOMO–LUMO gap (Table 4). Meanwhile, the molecular symmetry evolves from *C*_2*v*_ via *D*_2*h*_ to *D*_3*h*_, which governs the count of equivalent reactive sites within the series. The decreasing LUMO energy qualitatively aligns with the trend toward more negative LEAE values at major active sites, although LEAE additionally accounts for electronic relaxation upon electron attachment. As presented in Figure 6 and Figure S15, additional secondary nucleophilic reaction sites are different for the three species. These sites are situated on the exterior face of the C_12_ ring at the intermediate C–C bonds (1.37 Å) for C_12_-(CO)_2_, both faces of the C_12_ ring at the intermediate C–C bonds (1.37 Å) for C_12_-(CO)_4_, and both faces of the C_12_ ring at the C–C bonds of OCCO units for C_12_-(CO)_6_. And the corresponding values are −1.19 eV, −1.24 eV, and −1.38 eV, respectively. It is noteworthy that the OWDD isosurfaces of these positions are positive for the former two, and negative for the latter. Although both faces of the C_12_ ring at the C–C bonds of OCCO units for the three species exhibit certain nucleophilic reactivity, the negative OWDD isosurfaces indicate these sites are more susceptible to electrophilic reactivity.

It is noteworthy that the present conclusions only reflect intrinsic electronically controlled reactivity tendencies. Factors including solvent effects, steric hindrance, and kinetic reaction barriers are not considered, and thus the predicted reactivity trend cannot be directly equated to experimental reaction observations.

## 4. Conclusions

In this work, the structural, electronic, and reactive properties of planar C_12_–(CO)*_n_* (*n* = 2, 4, 6) were systematically characterized via multi-descriptor analyses.

First, the C_12_ skeleton exhibits pronounced bond-length alternation, with π-electrons localized at short bonds rather than delocalized over the macrocyclic ring. The decrease in AV1245 from 1.04 (*n* = 2) to 0.40 (*n* = 6), the increase in FLU-π from 0.498 to 0.773, and persistently negative HOMA values, consistently exclude global aromaticity. OCCO fusion suppresses π_in_ delocalization (AV1245-π_in_: 0.60 → 0.09) far more strongly than π_out_ (0.44 → 0.31), confirming that a single aromaticity index is insufficient for this class of functionalized π-macrocycles.

Second, simulated UV-Vis spectra show that increasing OCCO content shifts the dominant absorption bands toward the ultraviolet region; the strongest band for *n* = 6 appears at 280.7 nm. Hole–electron analyses verify that low-energy excitations are local excitations dominated by intra-ring charge redistribution. UFF-based vdW potential reveals an anomalous He > Ne adsorption strength sequence, and it differs from the BSSE-corrected DFT binding energy results obtained on fully optimized geometries.

Third, LEAE and OWDDs jointly establish the intrinsic electron-accepting reactivity order C_12_-(CO)_6_ > C_12_-(CO)_4_ > C_12_-(CO)_2_. The most negative LEAE values decrease progressively from −1.53 eV (*n* = 2) to −1.84 eV (*n* = 4) and −1.96 eV (*n* = 6). This trend arises from the cumulative electron-withdrawing effect of -CO groups. Molecular symmetry evolves from *C*_2*v*_ through *D*_2*h*_ to *D*_3*h*_ with increasing *n*, governing the number of equivalent reactive sites.

In summary, -CO substitution modulates reactivity intensity and the multiplicity of equivalent reactive sites in OCCO-fused macrocycles. This regulatory mechanism may be qualitatively transferable to analogous fused-ring systems, though further validation is required. This work may provide a helpful reference for synthesizing the C_12_ ring using C_12_–(CO)*_n_* (*n* = 2, 4, 6) as precursors.

## Figures and Tables

**Table 1:** AV1245-π multicenter indices (total, π_in_, and π_out_) and minimum multicenter indices (AV_min_-π, AV_min_-π_in_, AV_min_-π_out_) for C_12_ ring of C_12_-(CO)*_n_* (*n* = 2, 4 and 6).

	AV1245-π_in_	AV1245-π_out_	AV1245	AV_min_-π_in_	AV_min_-π_out_	AV_min_-π
C_12_-(CO)_2_	0.60 (0.51)	0.44 (2.18)	1.04 (2.69)	0.006	0.358	0.323
C_12_-(CO)_4_	0.14 (0.22)	0.42 (1.89)	0.56 (2.12)	0.016	0.107	0.229
C_12_-(CO)_6_	0.09 (−0.53)	0.31 (1.60)	0.40 (1.07)	0.013	0.153	0.317
C_18_	1.73	2.52	4.25	-	-	-

Note: The values in parentheses are the corresponding values for C_18_-(CO)*_n_* (*n* = 2, 4 and 6) [5].

**Table 2:** ELF-π bifurcation values (total, πin, and πout), HOMA indices, and FLU-π values for C_12_ ring of C_12_-(CO)*_n_* (*n* = 2, 4 and 6). (All values are dimensionless.)

	ELF-π_in_	ELF-π_out_	ELF-π	HOMA	FLU-π
C_12_-(CO)_2_	0.0065	0.415	0.187	−2.410	0.498
C_12_-(CO)_4_	0.0025	0.425	0.195	−1.866	0.652
C_12_-(CO)_6_	0.0012	0.425	0.288	−1.258	0.773

**Table 3:** Key electronic descriptors for selected excited states of C_12_-(CO)*_n_* (*n* = 2, 4, and 6) in the gas phase (HDI and EDI are dimensionless.).

*n*	State	Sr (a.u.)	*D* (Å)	*t* (Å)	*H* (Å)	HDI	EDI	E_coul_ (eV)	Excitation Types
2	S2	0.773	0.413	−1.828	2.886	6.57	6.23	5.32	LE
4	S8	0.857	0	−0.885	3.047	5.74	4.78	5.21	LE
6	S11	0.809	0.255	−2.270	3.458	5.65	5.52	4.51	LE
	S12	0.818	0.256	−1.939	3.458	5.65	5.52	4.52	LE

Note: Sr, *D*, *t*, *H*, HDI, EDI and E_coul_ denote Sr index (integral of Sr function), *D* index, *H* index, *t* index, hole delocalization index, Electron delocalization index and Coulomb attractive energy, respectively.

**Table 4:** Frontier-orbital energies (eV) for C_12_-(CO)*_n_* (*n* = 2, 4, 6).

	E_HOMO_ (eV)	E_LUMO_ (eV)	E_gap_ (eV)
C_12_-(CO)_2_	−8.69	−2.69	6.00
C_12_-(CO)_4_	−9.23	−3.26	5.97
C_12_-(CO)_6_	−9.47	−3.69	5.78

## Data Availability

The original contributions presented in this study are included in the article/Supplementary Materials. Further inquiries can be directed to the corresponding authors.

## Supplementary Materials

The following supporting information can be downloaded at https://www.mdpi.com/article/10.3390/nano16191254/s1, Table S1: Optimized Cartesian coordinates for C_12_-(CO)*_n_* (*n* = 2, 4, and 6) at the ωB97XD/def2-TZVP level; Figure S1: Cavity of C_12_-(CO)*_n_*; Figure S2: Numbering of chemical bonds of C_12_-(CO)*_n_*; Table S2: Bond orders of chemical bonds for C_12_-(CO)*_n_* calculated at the ωB97XD/def2-TZVP level; Figure S3: Optimized structures and related structural parameters of C_12_-(CO)*_n_* (*n* = 2, 4 and 6) at the CAM-B3LYP/def2-TZVP and PBE1PBE/def2-TZVP levels; Table S3: Comparison of bond lengths and bond angles of C_12_-(CO)*_n_* (*n* = 2, 4, and 6) calculated with the three functionals ωB97XD, CAM-B3LYP, and PBE1PBE using the def2-TZVP basis set; Table S4: Comparison of bond orders of C_12_-(CO)*_n_* (*n* = 2, 4, and 6) calculated with the three functionals ωB97XD, CAM-B3LYP, and PBE1PBE using the def2-TZVP basis set; Figure S4: IRI color bar; Figure S5: STM image of C_12_-(CO)*_n_*; Figure S6: Electron density difference and Gaussian-smoothed C^+^/C^−^ function isosurfaces for C_12_-(CO)_n_; Figure S7: Orbital interaction diagram of C_12_-(CO)*_n_*; Table S5: Contributions of fragment orbitals to frontier molecular orbitals of C_12_-(CO)*_n_*; Figure S8: Isosurface map of occupied π_in_ and π_out_ molecular orbitals of C_12_-(CO)*_n_*; Text S1. Physical meaning, thresholds, and limitations of the aromaticity descriptors; Figure S9: Isosurface contour of ELF-π_in_, ELF-π_out_, and ELF-π of C_12_-(CO)*_n_*; Figure S10: Simulated absorption spectra and charge-transfer spectra of C_12_–(CO)_2_ (200–715 nm and 200–425 nm); Table S6: Excited energy, excited wavelength, oscillator strength and main molecular orbital transitions of 20 excited states of C_12_-(CO)*_n_* at TD-ωB97XD/def2-TZVP level (based on the optimized structure at the level of ωB97XD/def2-TZVP); Table S7: Excited energy, excited wavelength, oscillator strength and main molecular orbital transitions of 20 excited states of C_12_-(CO)*_n_* at TD-CAM-B3LYP/def2-TZVP level (based on the optimized structure at the level of ωB97XD/def2-TZVP); Figure S11. Comparison of the simulated UV-Vis spectra of C_12_–(CO)*_n_* calculated at TD-CAM-B3LYP/def2-TZVP and TD-ωB97XD/def2-TZVP levels based on the same structure optimized at the ωB97XD/def2-TZVP level; Table S8: Excited energy, excited wavelength, oscillator strength and main molecular orbital transitions of low-lying excited states of C_12_–(CO)*_n_* obtained from TD-CAM-B3LYP/def2-TZVP and TD-ωB97XD/def2-TZVP levels; Figure S12: NTO pair for low-lying excited states of C_12_–(CO)*_n_*; Figure S13: Excited-state hole-electron analyses for C_12_-(CO)*_n_*; Text S2: Hole-electron analysis and assignment of locally excited states; Figure S14: Isosurface of the vdW potential of C_12_-(CO)*_n_* with various probes; Table S9: The van der Waals potential minima and vertical distances between noble gas atom and molecular plane for noble-gas probes over C_12_-(CO)*_n_* substrates; Table S10: SAPT0 energy decomposition results for the C_12_-(CO)_n_@He/Ne complex at the SAPT0/juncc-pVDZ level; Table S11: Raw interaction energies ΔEraw, counterpoise-corrected interaction energies ΔE_CP_, and BSSE values for C_12_-(CO)*_n_*@He/Ne (*n* = 2, 4, 6) calculated using different methods; Table S12: Vertical distances (Å) between the noble-gas atom and the C_12_ ring for C_12_-(CO)*_n_*@He/Ne (*n* = 2, 4, 6); Table S13: Optimized Cartesian coordinates for C_12_-(CO)*n*@He/Ne (*n* = 2, 4, 6) at the ωB97XD/def2-TZVP level; Figure S15: Isosurface maps of the orbital-weighted dual descriptor (OWDD) for C_12_-(CO)*_n_*; Text S3: Additional information on LEAE and OWDD.

## Abbreviations

The following abbreviations are used in this manuscript:

IRI	Interaction region indicator
LOL	Localized orbital locator
EDD	Electron density difference
ECDA	Extended charge decomposition analyses
HOMA	Harmonic oscillator measure of aromaticity
ELF	Electron localization function
FLU	Aromatic fluctuation index
CTS	Charge-transfer spectra
vdW	van der Waals
ESP	Electrostatic potential
OWDD	Orbital-weighted dual descriptor
LEAE	Local electron attachment energy
HOMO	The highest occupied orbital
LUMO	The lowest unoccupied orbital
UFF	Universal force field
SAPT	Symmetry adapted perturbation theory
BSSE	Basis set superposition error
