# Supplementary material for: Structural, Aromatic, Optical and Reactive Features of C12-(CO)n (n = 2, 4, 6): A DFT Study
Source: Nanomaterials (Basel). 2026 Oct 3;16(19):1254. doi: 10.3390/nano16191254 (PMC13666625; doi:10.3390/nano16191254)
Supplement: Supplementary file 1 [file nanomaterials-16-01254-s001.zip › nanomaterials-4562941-supplementary.pdf]

Table S1. Optimized Cartesian coordinates for C_{12}-$(CO)_n$ (n = 2, 4, and 6) at the ωB97XD/def2-TZVP level. The values are given in Å.

C_{12}-$(CO)_2$ (charge = 0, spin multiplicity = 1)			
Atom	x	y	z
C	0.00000000	1.53561600	-0.39169900
C	0.00000000	0.68270500	-1.51149500
C	0.00000000	-0.68270500	-1.51149500
C	0.00000000	-1.70755500	3.20493700
C	0.00000000	-2.14598800	2.07449900
C	0.00000000	-1.53561600	-0.39169900
C	0.00000000	-2.03033800	0.71103500
C	0.00000000	1.70755500	3.20493700
C	0.00000000	2.14598800	2.07449900
C	0.00000000	-0.60720100	4.02135900
C	0.00000000	0.60720100	4.02135900
C	0.00000000	2.03033800	0.71103500
C	0.00000000	0.78677200	-3.02094800
O	0.00000000	1.66199800	-3.81576700
C	0.00000000	-0.78677200	-3.02094800
O	0.00000000	-1.66199800	-3.81576700

C_{12}-$(CO)_4$ (charge = 0, spin multiplicity = 1)			
Atom	x	y	z
C	0.00000000	1.47767500	1.84761800
C	0.00000000	0.68309400	3.00568500
C	0.00000000	-0.68309400	3.00568500
C	0.00000000	-1.47767500	-1.84761800
C	0.00000000	-1.79693900	-0.68322600
C	0.00000000	-1.47767500	1.84761800
C	0.00000000	-1.79693900	0.68322600
C	0.00000000	1.47767500	-1.84761800
C	0.00000000	1.79693900	-0.68322600
C	0.00000000	-0.68309400	-3.00568500
C	0.00000000	0.68309400	-3.00568500
C	0.00000000	1.79693900	0.68322600
C	0.00000000	0.78947700	4.51647700
O	0.00000000	1.66259700	5.31135000
C	0.00000000	-0.78947700	4.51647700
O	0.00000000	-1.66259700	5.31135000
C	0.00000000	0.78947700	-4.51647700
C	0.00000000	-0.78947700	-4.51647700
O	0.00000000	1.66259700	-5.31135000
O	0.00000000	-1.66259700	-5.31135000

C_{12}-$(CO)_6$ (charge = 0, spin multiplicity = 1)			
Atom	x	y	z
C	0.60185600	2.13789300	0.00000000
C	1.98921800	1.93465500	0.00000000
C	2.67006900	0.75538600	0.00000000
C	-0.68085100	-2.69004100	0.00000000
C	0.68085100	-2.69004100	0.00000000
C	2.15239800	-0.54772400	0.00000000
C	1.55054200	-1.59016900	0.00000000
C	-2.67006900	0.75538600	0.00000000
C	-1.98921800	1.93465500	0.00000000
C	-1.55054200	-1.59016900	0.00000000
C	-2.15239800	-0.54772400	0.00000000
C	-0.60185600	2.13789300	0.00000000
C	3.24746200	2.78595300	0.00000000
O	3.49402300	3.93944200	0.00000000
C	4.03643700	1.41940800	0.00000000
O	5.15866800	1.05619200	0.00000000
C	-3.24746200	2.78595300	0.00000000
C	-4.03643700	1.41940800	0.00000000
O	-3.49402300	3.93944200	0.00000000
O	-5.15866800	1.05619200	0.00000000
C	-0.78897500	-4.20536200	0.00000000
C	0.78897500	-4.20536200	0.00000000
O	-1.66464500	-4.99563400	0.00000000
O	1.66464500	-4.99563400	0.00000000

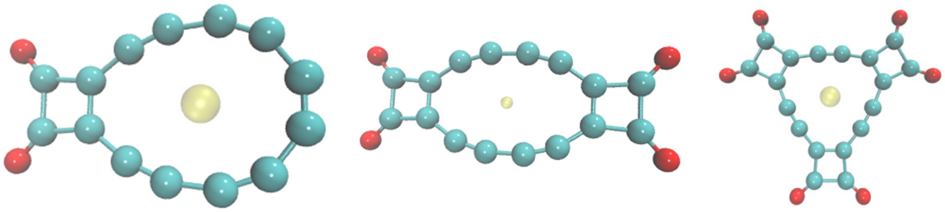

Figure S1. Cavity of C_{12}-$(CO)_n$ (n = 2, 4, and 6, their C_{12} ring cavity diameters are 1.004 Å, 0.444 Å, and 1.042 Å, respectively.) Cyan and red spheres represent carbon atoms and oxygen atoms, respectively. The pale yellow sphere indicates the cavity inside the ring.

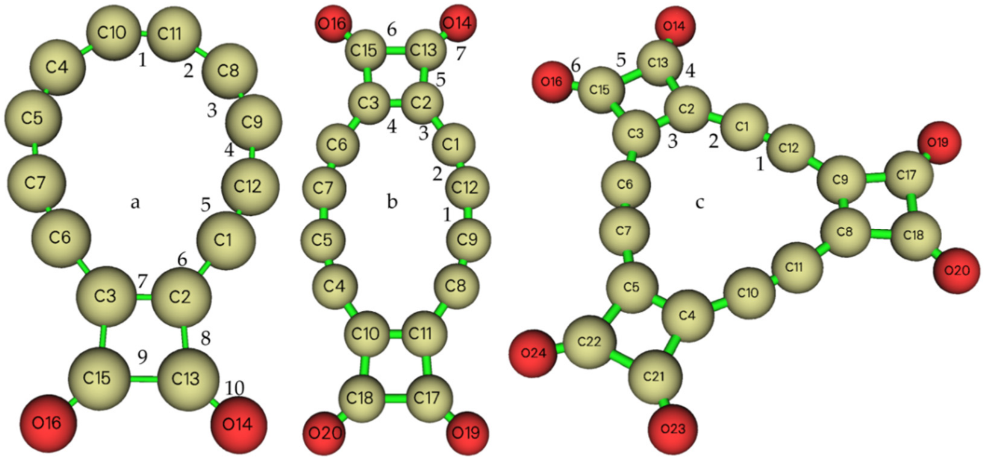

Figure S2. Numbering of atoms and chemical bonds of C_{12}-$(CO)_n$ (n = 2, 4, and 6.) Light brown spheres represent carbon atoms, red spheres denote oxygen atoms, and green sticks stand for chemical bonds.

Table S2. Bond orders (Mayer, Wiberg, Mulliken, Fuzzy and Laplacian) of chemical bonds for C_{12}-$(CO)_n$ (n = 2, 4, and 6 calculated at the ωB97XD/def2-TZVP level. Numbering of chemical bonds of C_{12}-$(CO)_n$ are shown in **Figure S2**.)

	No. of chemical bonds	Mayer	Wiberg	Mulliken	Fuzzy	Laplacian
C_{12}-$(CO)_2$	1	2.54	2.45	1.78	2.38	2.52
	2	1.18	1.43	0.92	1.34	1.51
	3	2.61	2.46	1.82	2.39	2.53
	4	1.13	1.43	0.80	1.34	1.58
	5	2.72	2.52	1.20	2.44	2.56
	6	1.10	1.29	0.63	1.21	1.45
	7	1.54	1.50	1.15	1.45	1.59
	8	0.89	1.06	-0.10	1.00	0.98
	9	0.97	0.99	0.78	0.90	0.86
	10	2.14	2.79	1.30	2.16	1.41
C_{12}-$(CO)_4$	1	1.16	1.42	0.70	1.33	1.62
	2	2.74	2.51	1.46	2.43	2.57
	3	1.07	1.29	0.74	1.22	1.45
	4	1.48	1.48	1.02	1.44	1.57
	5	0.87	1.06	-0.17	1.00	0.97
	6	0.98	0.98	0.75	0.89	0.85
	7	2.15	2.79	1.29	2.16	1.42
C_{12}-$(CO)_6$	1	2.77	2.56	-0.07	2.48	2.60
	2	1.08	1.29	0.39	1.22	1.49

	3	1.52	1.51	1.01	1.46	1.61
	4	0.89	1.05	-0.08	0.99	0.96
	5	0.98	0.98	0.74	0.89	0.85
	6	2.16	2.80	1.29	2.17	1.43

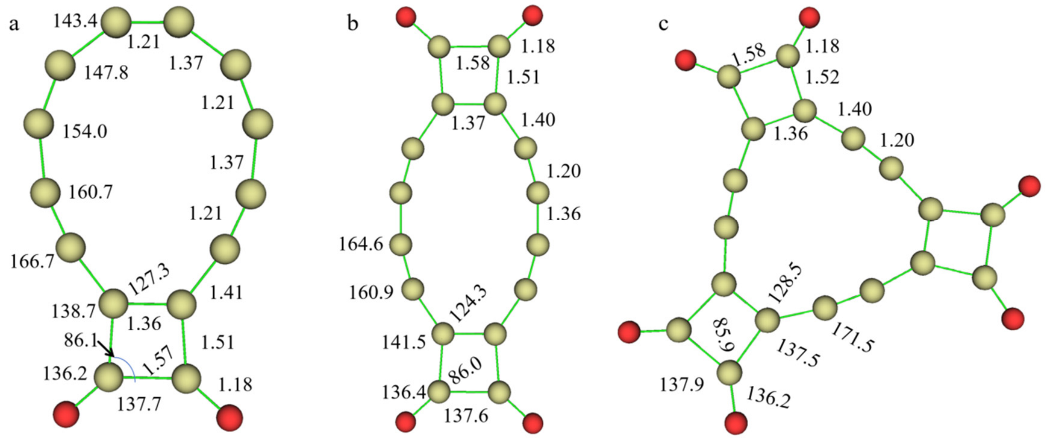

Figure S3(a). Optimized structures and related structural parameters of C_{12}-$(CO)_n$ (n = 2, 4 and 6) at the CAM-B3LYP/def2-TZVP level. Bond angles (left) are given in ° and bond lengths (right) are given in Å (Light brown spheres: C; red spheres: O. Green lines: chemical bonds.).

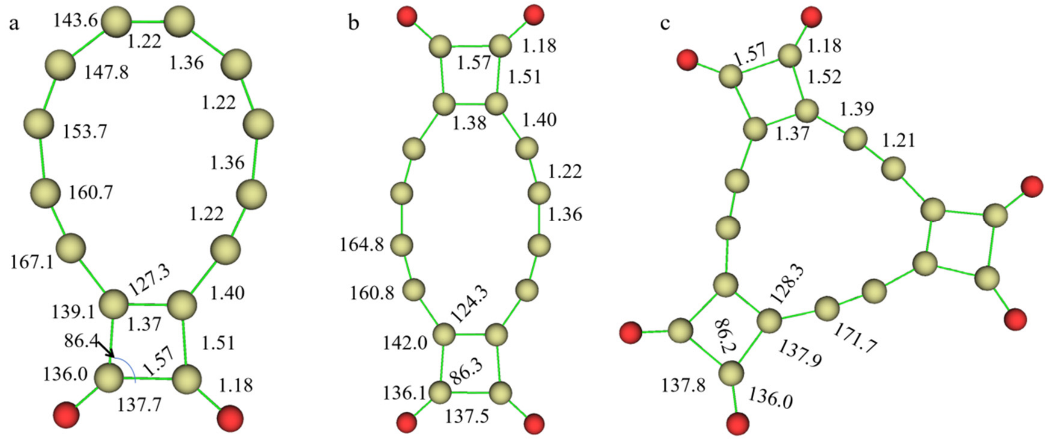

Figure S3(b). Optimized structures and related structural parameters of C_{12}-$(CO)_n$ (n = 2, 4 and 6) at the PBE1PBE/def2-TZVP level. Bond angles (left) are given in ° and bond lengths (right) are given in Å (Light brown spheres: C; red spheres: O. Green lines: chemical bonds.).

Table S3(a). Comparison of bond lengths (Å) of C_{12}-$(CO)_n$ (n = 2, 4, and 6) calculated with the three functionals ωB97XD, CAM-B3LYP, and PBE1PBE using the def2-TZVP basis set.

	bond	ωB97XD	cam-b3lyp	pbe1pbe
C_{12}-$(CO)_2$	1	1.21	1.21	1.22
	2	1.37	1.37	1.36
	3	1.21	1.21	1.22

	4	1.37	1.37	1.36
	5	1.21	1.21	1.22
	6	1.41	1.41	1.40
	7	1.37	1.36	1.37
	8	1.51	1.51	1.51
	9	1.57	1.57	1.57
	10	1.18	1.18	1.18
C_{12}-$(CO)_4$	1	1.37	1.36	1.36
	2	1.21	1.20	1.22
	3	1.40	1.40	1.40
	4	1.37	1.37	1.38
	5	1.51	1.51	1.51
	6	1.58	1.58	1.57
	7	1.18	1.18	1.18
C_{12}-$(CO)_6$	1	1.20	1.20	1.21
	2	1.40	1.40	1.39
	3	1.36	1.36	1.37
	4	1.52	1.52	1.52
	5	1.58	1.58	1.57
	6	1.18	1.18	1.18

Note: The bond numbering follows that in Figure S2. PBE1PBE corresponds to PBE0.

Table S3(b). Comparison of bond angles (Å) of C_{12}-$(CO)_n$ (n = 2, 4, and 6) calculated with the three functionals ωB97XD, CAM-B3LYP, and PBE1PBE using the def2-TZVP basis set.

	Bond angle	ωB97XD	cam-b3lyp	pbe1pbe
C_{12}-$(CO)_2$	C13-C15-O16	137.8	137.7	137.7
	C13-C15-C3	86.1	86.1	86.4
	C3-C15-O16	136.2	136.2	136.0
	C15-C3-C6	138.8	138.7	139.1
	C2-C3-C6	127.3	127.4	127.2
	C3-C6-C7	166.9	166.7	167.1
	C6-C7-C5	160.7	160.7	160.7
	C7-C5-C4	154.0	154.0	153.7
	C5-C4-C10	147.8	147.8	147.8
	C4-C10-C11	143.4	143.4	143.6
C_{12}-$(CO)_4$	C17-C18-O20	137.7	137.6	137.5
	C17-C18-C10	86.0	86.0	86.3
	C10-C18-O20	136.3	136.4	136.1
	C11-C10-C4	124.5	124.5	124.3
	C18-C10-C4	141.5	141.5	142.0
	C10-C4-C5	160.9	160.9	160.8
	C4-C5-C7	164.7	164.6	164.8
C_{12}-$(CO)_6$	C22-C21-O23	137.9	137.9	137.8

	C4-C21-O23	136.1	136.2	136.0
	C22-C21-C4	85.9	85.9	86.2
	C21-C4-C10	137.6	137.5	137.9
	C5-C4-C10	128.3	128.5	128.3
	C4-C10-C11	171.7	171.5	171.7

Note: The numbering of atoms of C_{12}-$(CO)_n$ (n = 2, 4, and 6.) are shown in Figure S2.

Table S4. Comparison of Mayer bond orders of chemical bond of C_{12}-$(CO)_n$ (n = 2, 4, and 6) calculated with the three functionals ωB97XD, CAM-B3LYP, and PBE1PBE using the def2-TZVP basis set.

	bond	ωB97XD	cam-b3lyp	pbe1pbe
C_{12}-$(CO)_2$	1	2.54	2.52	2.45
	2	1.18	1.17	1.25
	3	2.61	2.62	2.44
	4	1.13	1.09	1.22
	5	2.72	2.88	2.50
	6	1.10	1.08	1.16
	7	1.54	1.48	1.50
	8	0.89	0.89	0.88
	9	0.97	0.98	0.94
	10	2.14	2.22	2.17
C_{12}-$(CO)_4$	1	1.16	1.08	1.29
	2	2.74	2.85	2.52
	3	1.07	1.10	1.11
	4	1.48	1.44	1.47
	5	0.87	0.88	0.86
	6	0.98	0.98	0.94
	7	2.15	2.23	2.18
C_{12}-$(CO)_6$	1	2.77	2.85	2.54
	2	1.08	1.10	1.14
	3	1.52	1.44	1.47
	4	0.89	0.88	0.87
	5	0.98	0.98	0.95
	6	2.16	2.23	2.19

Note: The bond numbering follows that in Figure S2. PBE1PBE corresponds to PBE0.

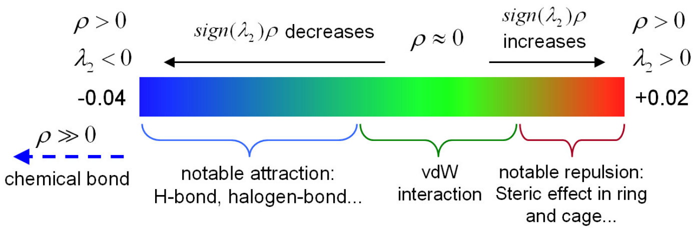

Figure S4. IRI color bar (sourced from Multiwfn code)

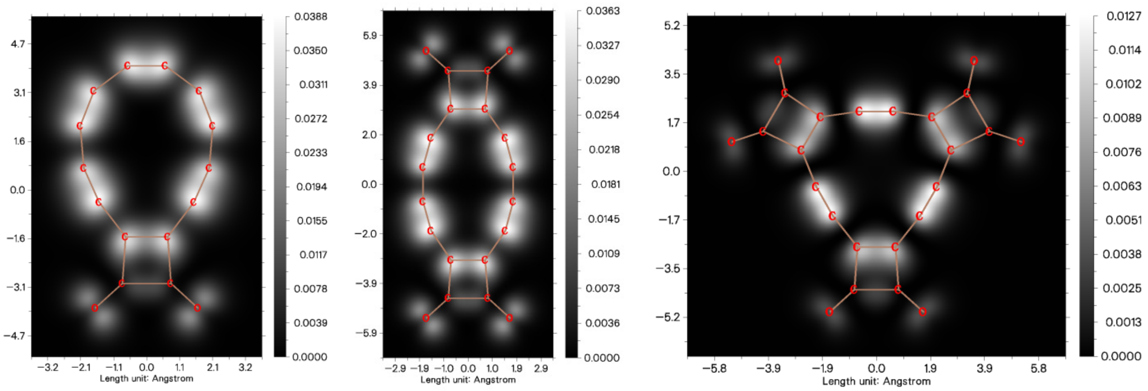

Figure S5. STM (Scanning Tunneling Microscopy) image of C_{12}-$(CO)_n$ (n = 2, 4, and 6) with a bias voltage of -3.5 V. The scale of color bar is in a.u.

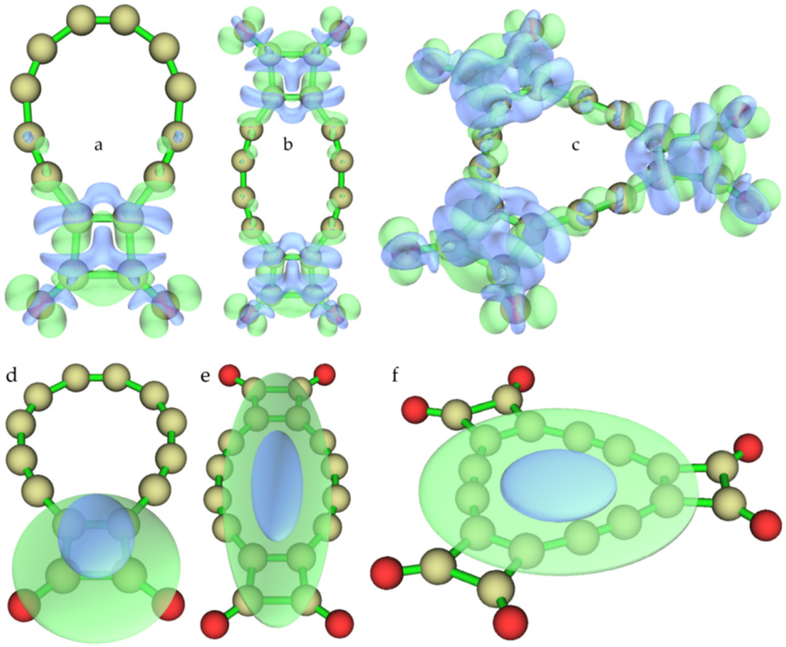

Figure S6. Electron density difference (a-c, isovalue = 0.006 a.u.) and Gaussian-smoothed C^+/C^- function isosurfaces (c-e, isovalue = 0.005 a.u.) for C_{12}-$(CO)_n$ (n = 2 for a and d, n = 4 for b and e, n = 6 for c and f.). For EDD, the green and blue parts represent the regions where electron density is increased and decreased. For smoothed C^+/C^- functions, green and blue isosurfaces characterize the regions of $C^+(\varrho^+)$ and $C^-(\varrho^-)$, respectively.

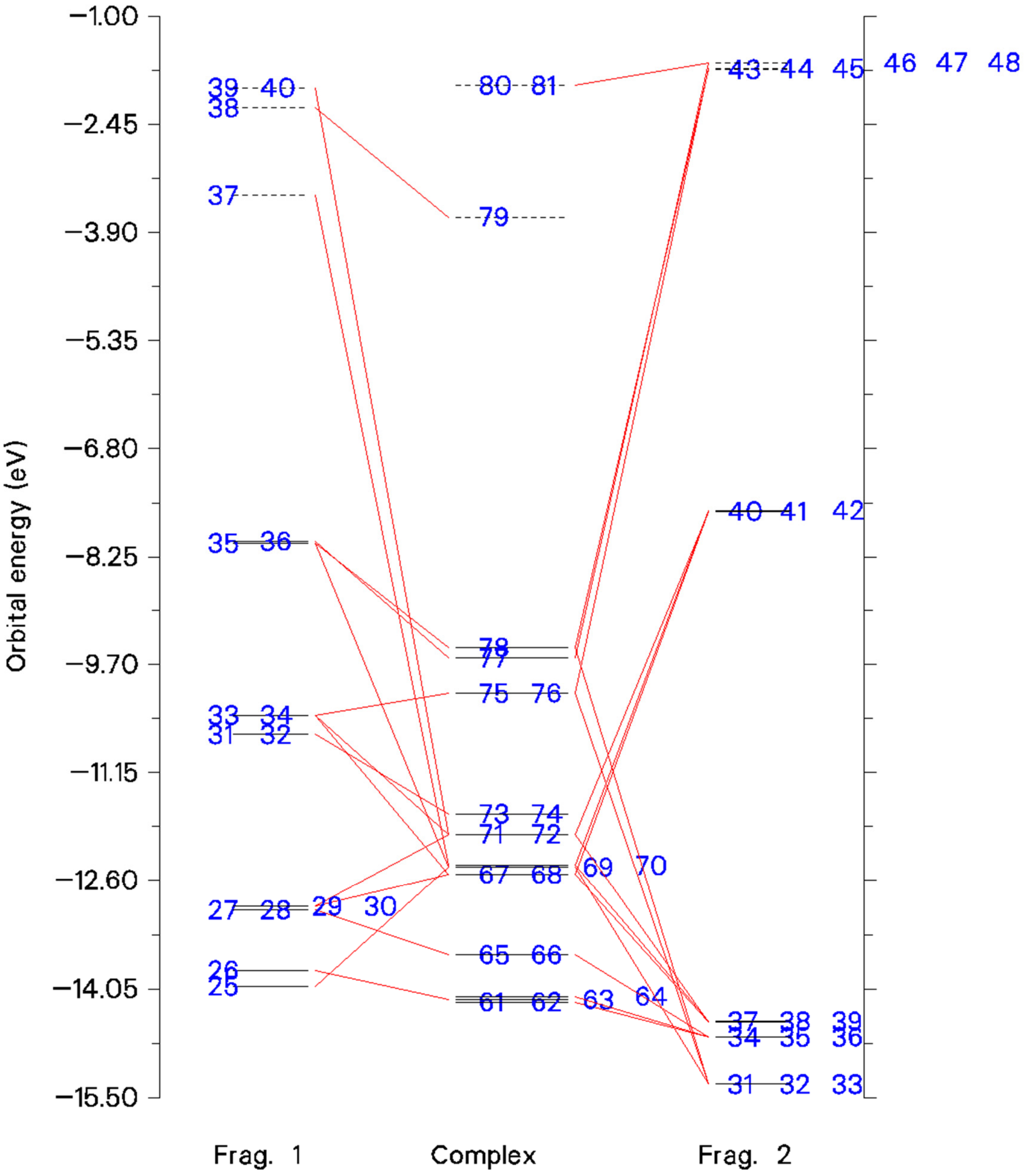

Figure S7(a). Orbital interaction diagram of C_{12}-$(CO)_6$. Black solid and black dashed bars correspond to occupied and unoccupied MOs, respectively. Blue texts mark orbital indices. Complex, Frag. 1 and Frag. 2 denote C_{12}-$(CO)_6$, C_{12} and $(CO)_6$, respectively.

Table S5(a). Contributions of fragment orbitals to frontier molecular orbitals of C_{12}-$(CO)_6$ (corresponding to **Figure S2(a)**, isovalue = 0.05 a.u.) The orbital compositions were evaluated by Mulliken method.

C_{12}-$(CO)_6$	C_{12}	$(CO)_6$	$(CO)_6$
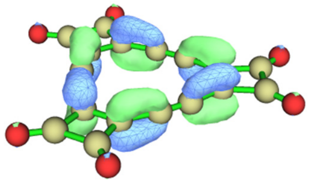<br>LUMO 79	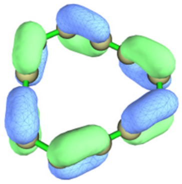<br>38 (89.97%)	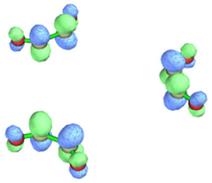<br>51 (8.21%)	
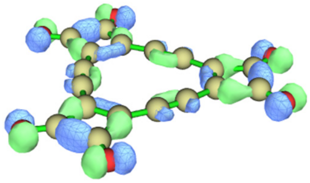<br>HOMO 78	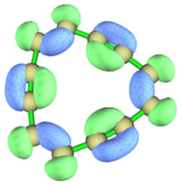<br>35 (38.27%)	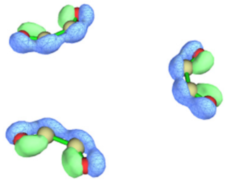<br>31 (25.22%)	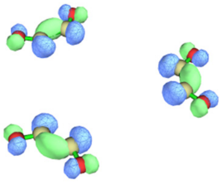<br>43 (31.22%)
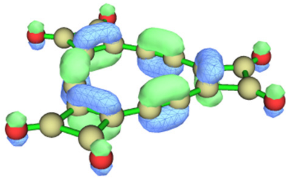<br>77	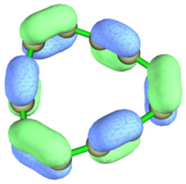<br>36 (79.07%)	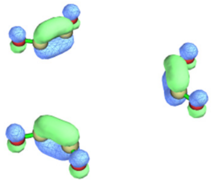<br>46 (12.32%)	
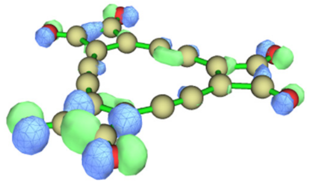<br>76	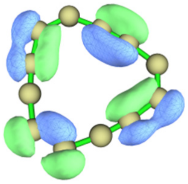<br>34 (28.47%)	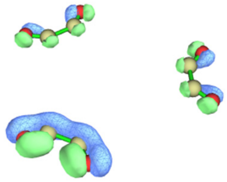<br>32 (37.81%)	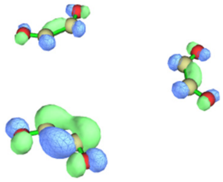<br>44 (26.27%)
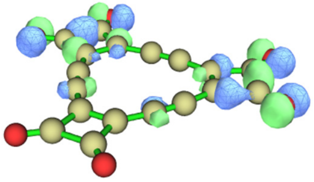<br>75	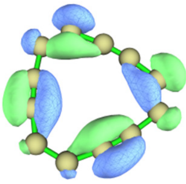<br>33 (28.47%)	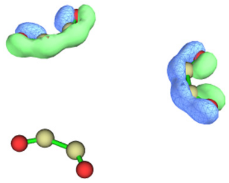<br>33 (37.81%)	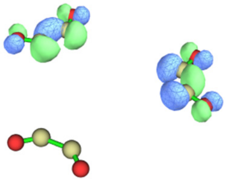<br>45 (26.27%)

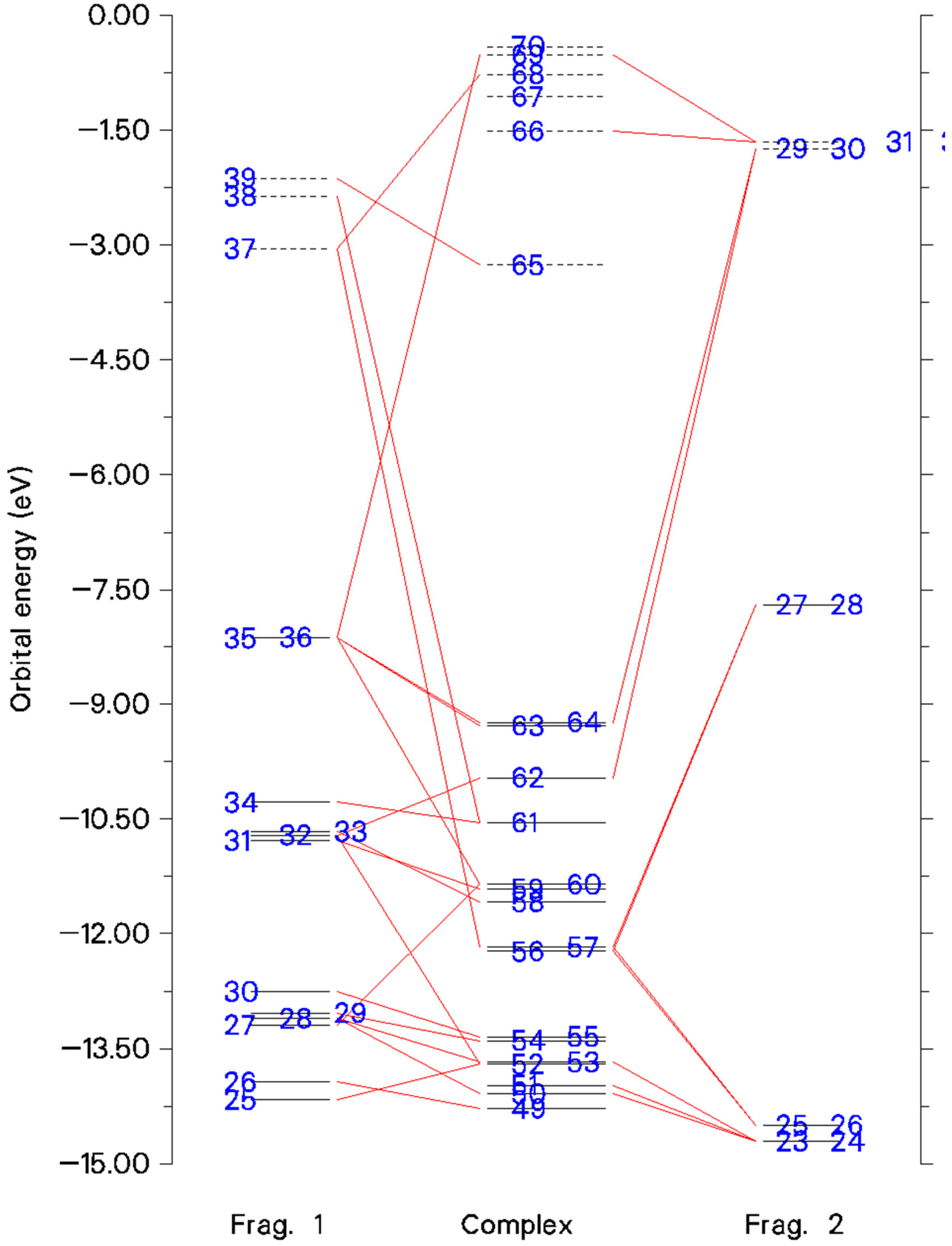

Figure S7(b). Orbital interaction diagram of C_{12}-$(CO)_4$. Black solid and black dashed bars correspond to occupied and unoccupied MOs, respectively. Blue texts mark orbital indices. Complex, Frag. 1 and Frag. 2 denote C_{12}-$(CO)_4$, C_{12} and $(CO)_4$, respectively.

Table S5(b). Contributions of fragment orbitals to frontier molecular orbitals of C_{12}-$(CO)_4$ (corresponding to Figure S2(b), isovalue = 0.05 a.u.) The orbital compositions were evaluated by Mulliken method.

C_{12}-$(CO)_4$	C_{12}	$(CO)_4$	$(CO)_4$
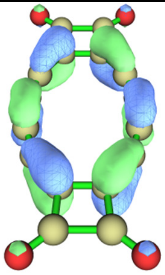<br>LUMO 65	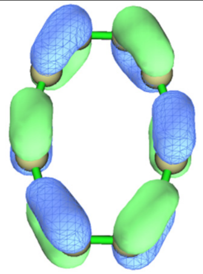<br>39 (91.02%)	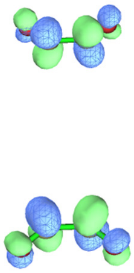<br>33 (7.17%)	
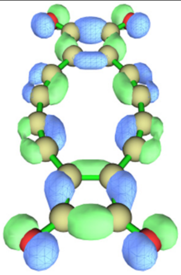<br>HOMO 64	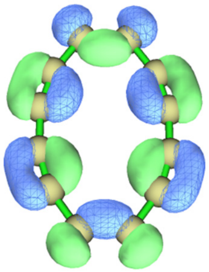<br>35 (51.42%)	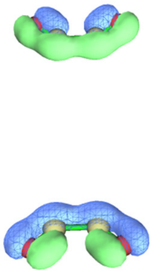<br>22 (21.31%)	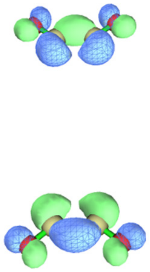<br>30 (24.45%)
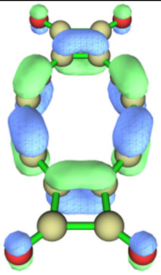<br>63	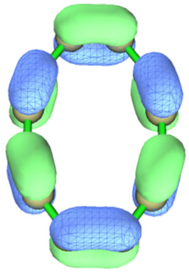<br>36 (83.90%)	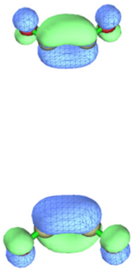<br>32 (9.36%)	
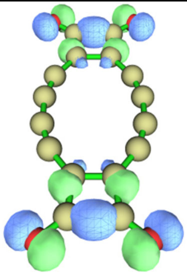<br>62	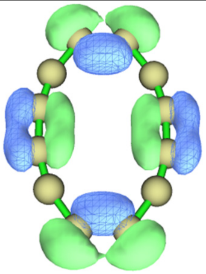<br>32 (25.02%)	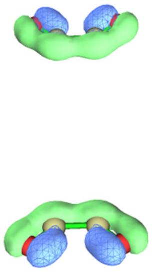<br>21 (41.24%)	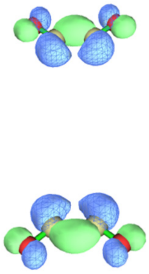<br>29 (28.81%)
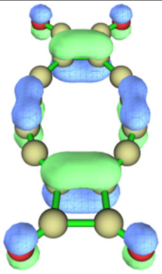<br>58	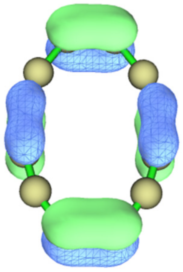<br>33 (77.80%)	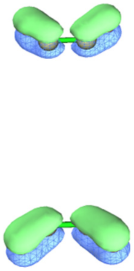<br>19 (15.41%)	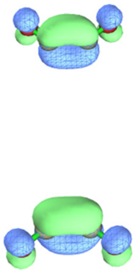<br>31 (6.69%)

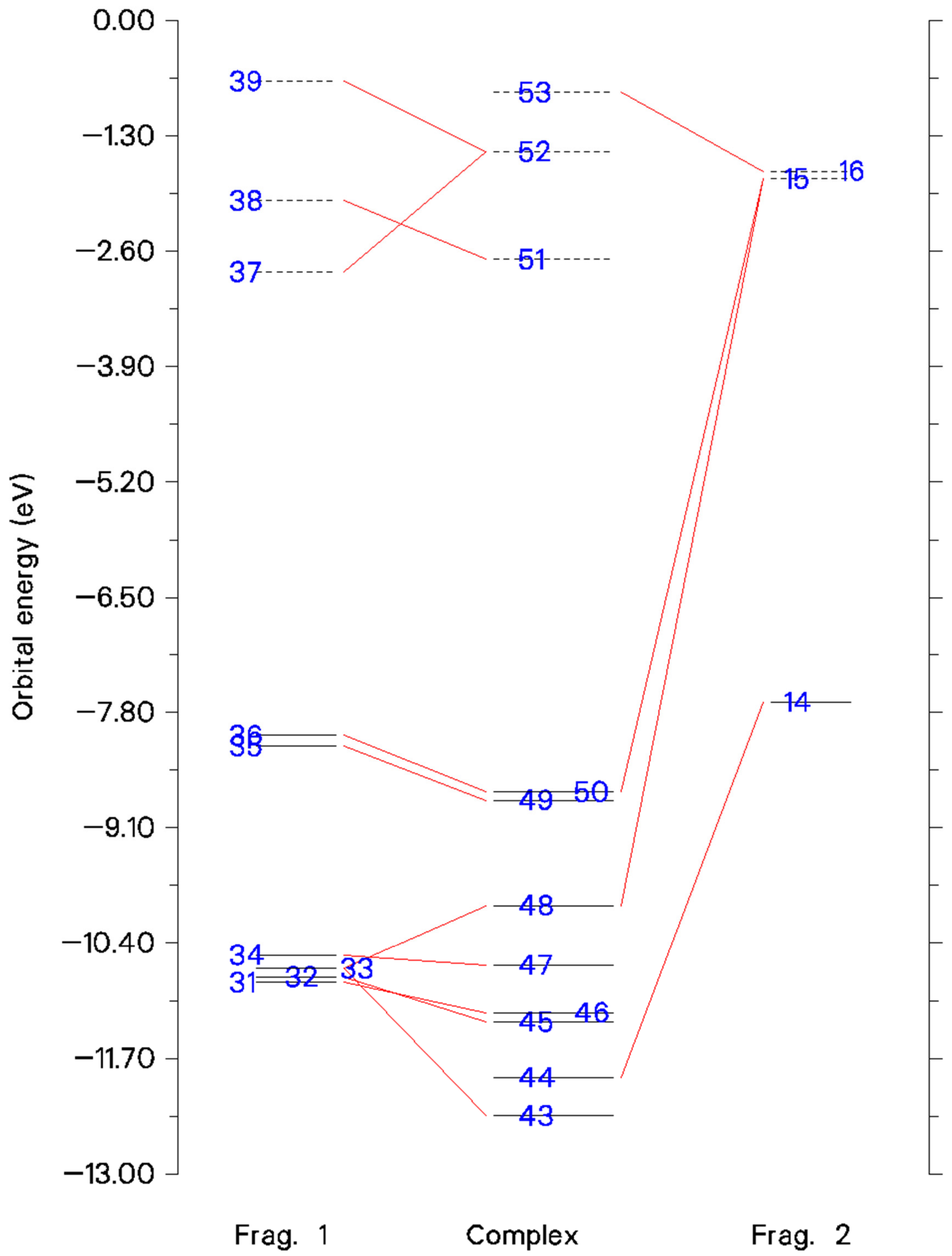

Figure S7(c). Orbital interaction diagram of C_{12}-$(CO)_2$. Black solid and black dashed bars correspond to occupied and unoccupied MOs, respectively. Blue texts mark orbital indices. Complex, Frag. 1 and Frag. 2 denote C_{12}-$(CO)_2$, C_{12} and $(CO)_2$, respectively.

Table S5(c). Contributions of fragment orbitals to frontier molecular orbitals of C_{12}-$(CO)_2$ (corresponding to Figure S2(c), isovalue = 0.05 a.u.) The orbital compositions were evaluated by Mulliken method.

C_{12}-$(CO)_2$	C_{12}	$(CO)_2$	$(CO)_2$
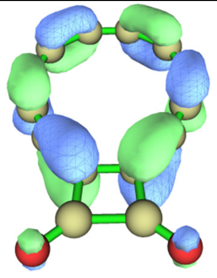 LUMO 51	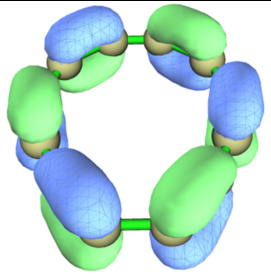 38 (92.42%)	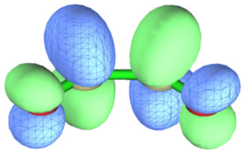 17 (5.44%)	
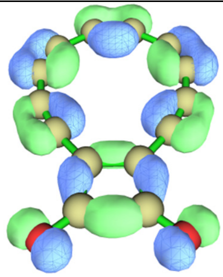 HOMO 50	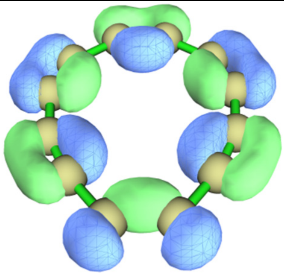 36 (77.04%)	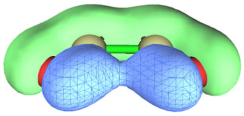 11 (7.99%)	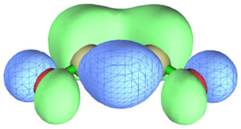 15 (11.74%)
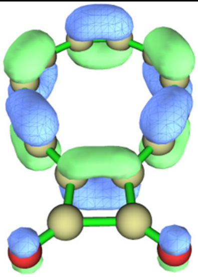 49	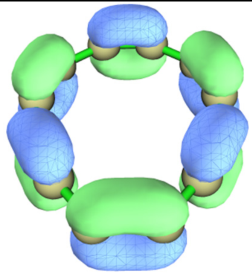 35 (90.65%)	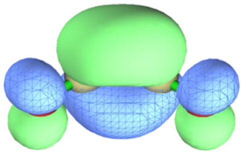 16 (4.83%)	
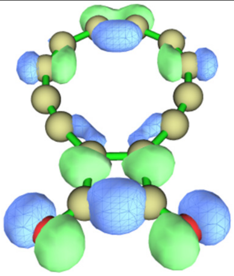 48	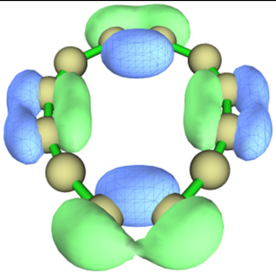 33 (36.07%)	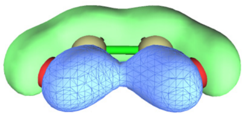 11 (32.24%)	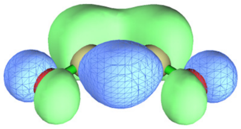 15 (17.51%)
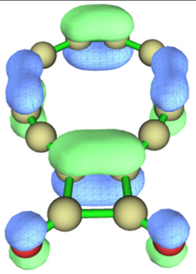 45	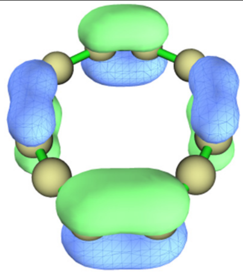 32 (87.29%)	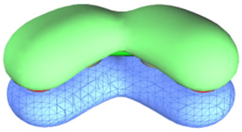 10 (8.50%)	

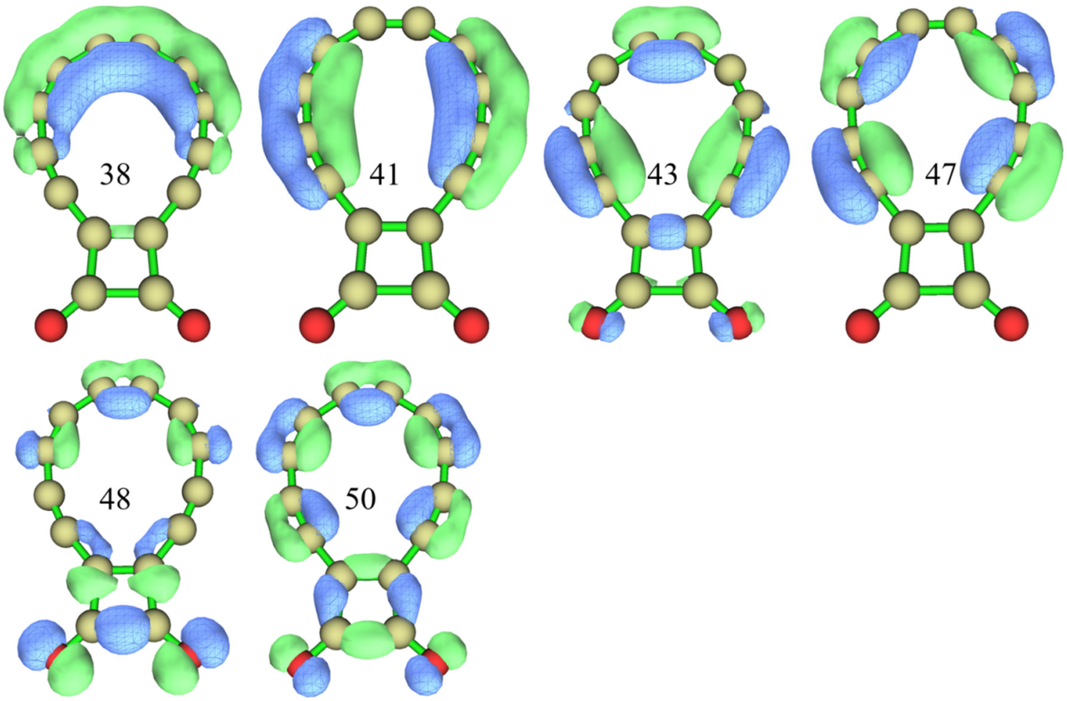

Figure S8(a). Isosurface map of occupied π_{in} molecular orbitals (isovalue = 0.05 a.u.) for C_{12}-$(CO)_2$. Green and blue regions denote positive and negative orbital phases, respectively. Orbital indices are labelled.

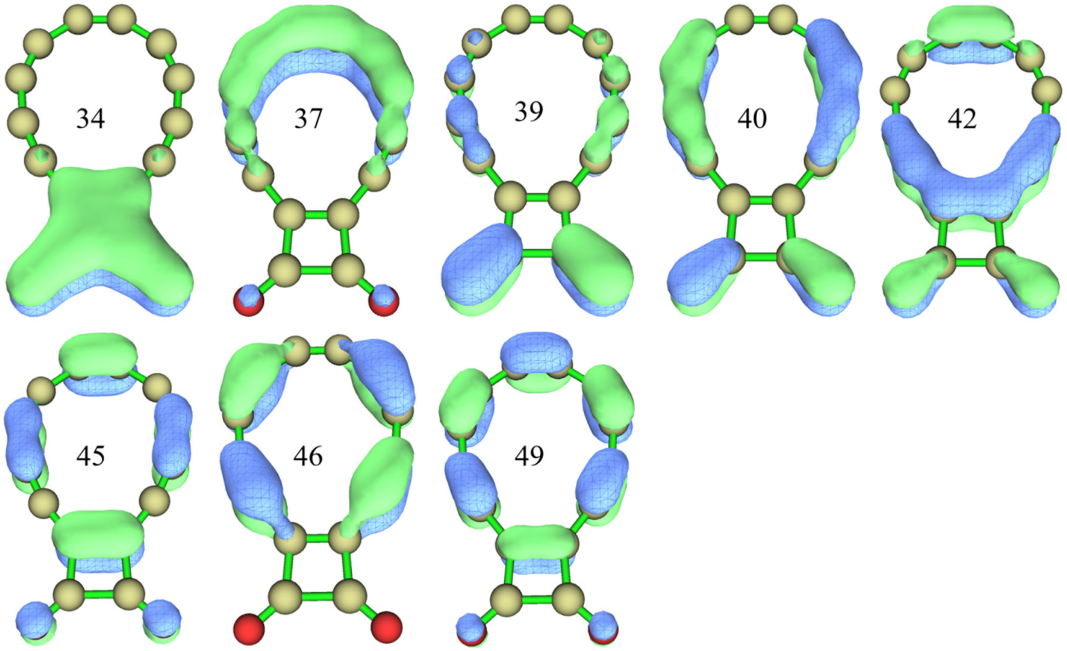

Figure S8(b). Isosurface map of occupied π_{out} molecular orbitals (isovalue = 0.05 a.u.) for C_{12}-$(CO)_2$. Green and blue regions denote positive and negative orbital phases, respectively. Orbital indices are labelled.

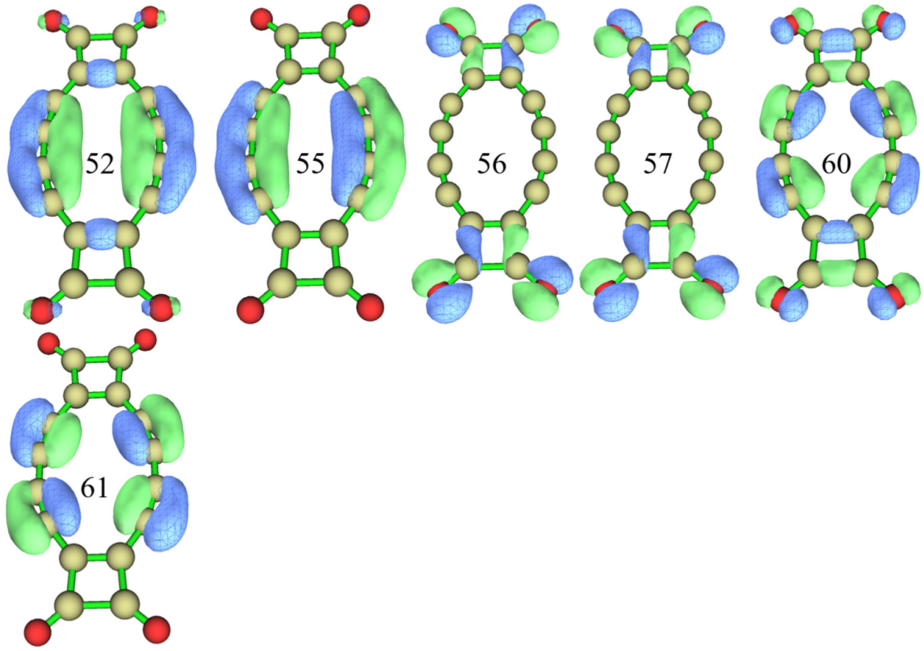

Figure S8(c). Isosurface map of occupied π_{in} molecular orbitals (isovalue = 0.05 a.u.) for C_{12}-$(CO)_4$. Green and blue regions denote positive and negative orbital phases, respectively. Orbital indices are labelled.

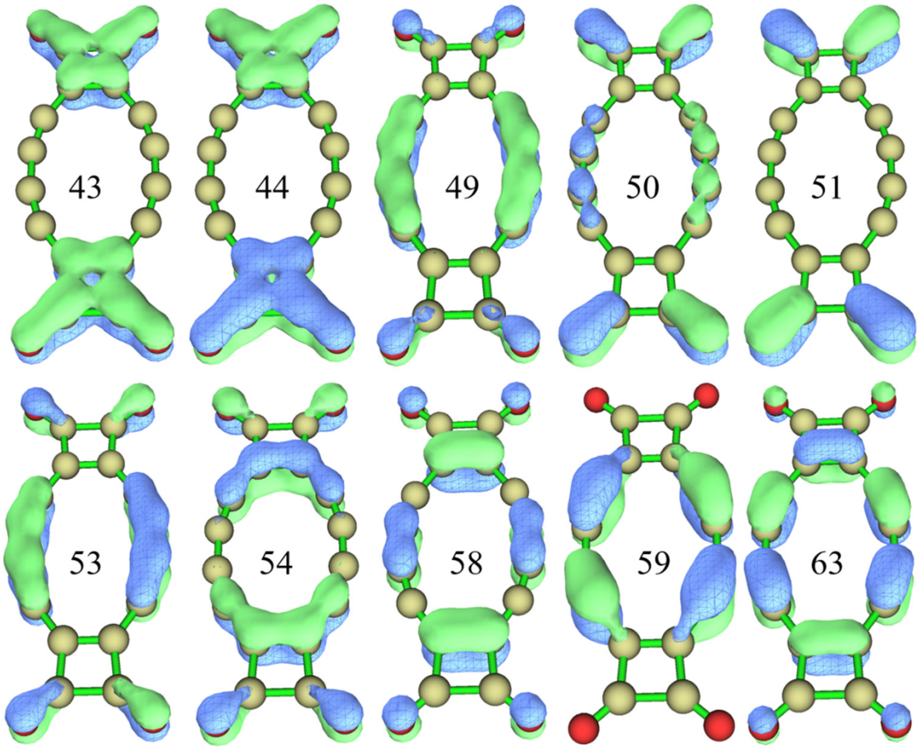

Figure S8(d). Isosurface map of occupied π_{out} molecular orbitals (isovalue = 0.05 a.u.) for C_{12}-$(CO)_4$. Green and blue regions denote positive and negative orbital phases, respectively. Orbital indices are labelled.

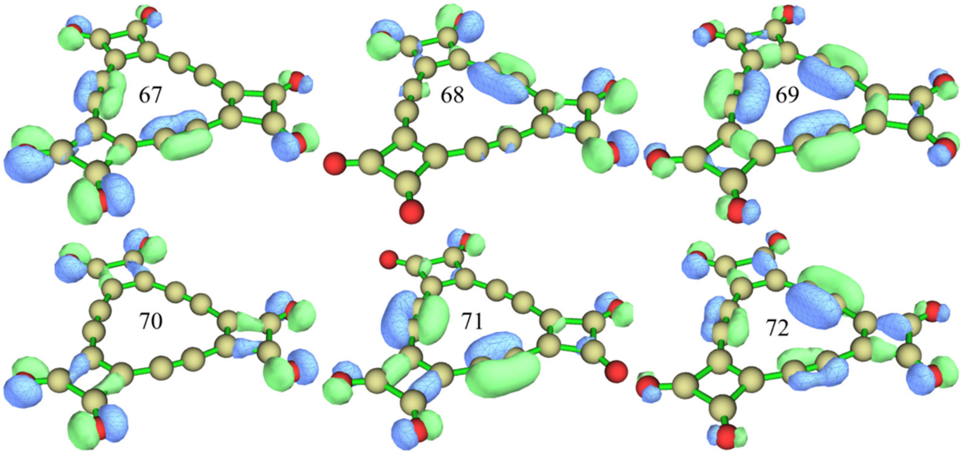

Figure S8(e). Isosurface map of occupied π_{in} molecular orbitals (isovalue = 0.05 a.u.) for C_{12}-$(CO)_6$. Green and blue regions denote positive and negative orbital phases, respectively. Orbital indices are labelled.

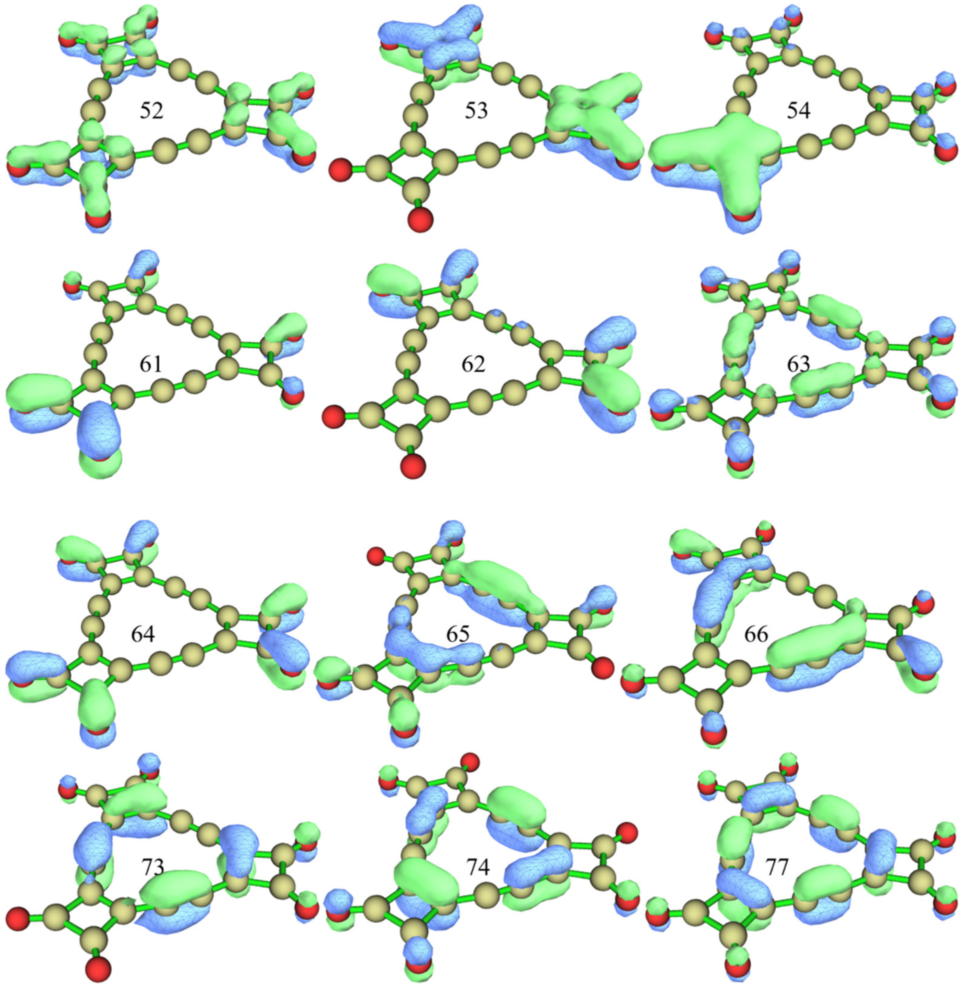

Figure S8(f). Isosurface map of occupied π_{out} molecular orbitals (isovalue = 0.05 a.u.) for C_{12}-$(CO)_6$. Green and blue regions denote positive and negative orbital phases, respectively. Orbital indices are labelled.

Text S1. Physical meaning, thresholds, and limitations of the aromaticity descriptors

Four descriptors were employed to assess aromaticity. Each probes a different physical aspect of aromaticity, and each has intrinsic limitations. Their combined use is therefore intended to reduce single metric bias rather than to provide a formal proof.

HOMA (Harmonic Oscillator Model of Aromaticity) [45,49,50] is a geometry-based index that measures bond-length equalization in a cyclic framework. HOMA = 1 corresponds to a fully aromatic ring in which all bonds equal the optimal reference lengths, HOMA = 0 corresponds to a completely nonaromatic ring, and significantly negative values indicate antiaromatic character. Thus, the closer HOMA is to 1, the stronger the aromaticity. Its main limitation is that it is purely geometric: it reflects structural homogeneity but does not directly measure π-electron delocalization, and its values depend on the reference bond lengths and computational method, as discussed by Andrzejak et al. in Struct. Chem. 2013, 24, 1171–1184 [50].

FLU-π (π contribution to the Aromatic Fluctuation Index, FLU) measures the fluctuation of electron sharing between adjacent atoms in a ring. FLU was proposed by Matito, Duran, and Solà in J. Chem. Phys. 2005, 122, 014109 [48]; see also Chem. Rev. 2005, 105, 3911–3947 [51] for further discussion. FLU-π is close to zero for aromatic species and becomes larger for nonaromatic or antiaromatic species; therefore, the closer FLU-π is to zero, the stronger the local π delocalization. For the π contribution, reference δ values are not required because δ ref is replaced with the average value of δ-π. Its limitation is that it is a local pairwise descriptor and provides no direct information on global cyclic conjugation. In addition, no universal numerical threshold has been established for FLU-π, so its values must be interpreted qualitatively or by comparison with suitable reference systems.

ELF-π (π contribution to the Electron Localization Function, ELF) is a real-space index. The separation of ELF into σ and π components was reported in J. Chem. Phys. 2004, 120, 1670–1673 [52] and J. Chem. Theory Comput. 2005, 1, 83–88 [53]. It is defined as the ELF value at the bifurcation point of π-type disynaptic basins. According to Poater et al. in Chem. Rev. 2005, 105, 3911–3947 [51], ELF-π > 0.70 is associated with π aromaticity. When the average of ELF-π and ELF-σ (σ contribution to ELF) exceeds 0.70, the molecule can be regarded as globally aromatic. Its limitation is that these thresholds are empirical, and values below 0.70 should be interpreted with caution because delocalization based indices cannot unambiguously separate nonaromatic from antiaromatic cases.

AV1245 (average of the four-center multicenter index, MCI, values at positions 1, 2, 4, and 5 along the ring perimeter) is designed to quantify cyclic multicenter π-electron delocalization

along a predefined ring pathway. It was proposed by Matito in Phys. Chem. Chem. Phys. 2016, 18, 11839–11846 [47] for large rings where conventional multicenter indices become numerically unstable. AV1245 is defined as the average of the 4-center MCI values along the ring that keep a positional relationship of 1, 2, 4, and 5, thereby measuring the extent of electron delocalization transferability between bonds 1–2 and 4–5. Larger AV1245 values indicate stronger aromaticity; benzene is characterized by AV1245 ≈ 10.5–10.71 depending on the level of theory. AV1245 has no universal absolute threshold and must be compared with appropriate reference systems. In addition, because AV1245 is an average along the ring, it may hide local failures of delocalization; AVmin (minimum absolute value of the four-center MCI along the ring) was proposed in J. Phys. Chem. C 2017, 121, 27118 and further discussed in Phys. Chem. Chem. Phys. 2018, 20, 2787–2796 [54,55], and provides a more sensitive measure of local delocalization because it is not based on average values.

Table. Summary of π-aromaticity criteria for the four descriptors.

Descriptor	Aromatic	Nonaromatic / Antiaromatic	Remarks
HOMA	≈ 1	≈ 0 (nonaromatic); significantly negative (antiaromatic)	Geometry-based; depends on reference bond lengths and method
FLU-π	≈ 0	> 0 (both nonaromatic and antiaromatic; cannot distinguish)	Local pairwise; no universal numerical threshold
ELF-π	> 0.70	< 0.70 cannot unambiguously separate nonaromatic from antiaromatic	Empirical; global aromaticity if average of ELF-π and ELF-σ > 0.70
AV1245	Larger values; benzene ≈ 10.5–10.71	Smaller values; cannot reliably distinguish antiaromatic	No absolute threshold; requires reference comparison; AVmin more sensitive

Application to the C_{12} ring: For the C_{12} ring in C_{12}-$(CO)_n$ (n = 2, 4, and 6), the four descriptors consistently indicate negligible π-aromaticity. HOMA is strongly negative (−2.410, −1.866, and −1.258 for n = 2, 4, and 6, respectively), far from the aromatic threshold 1, indicating pronounced bond-length alternation. FLU-π is large (0.498, 0.652, and 0.773), well above aromatic threshold 0 and consistent with nonaromatic or antiaromatic character. ELF-π is low (0.187, 0.195, and 0.288), far below the aromatic threshold 0.70. In particular, ELF-π_{in} is nearly zero (0.0012–0.0065), whereas ELF-π_{out} is 0.415–0.425, indicating that π localization occurs mainly outside the C_{12} ring rather than along the ring pathway. AV1245 is only 0.40–1.04, much lower than the benzene reference value 10.5–10.7, and AV_{min}-π_{in} is only 0.006–0.016, further indicating very weak multicenter π delocalization along the C_{12} ring.

Why the combined use supports the conclusion? These four descriptors are complementary: HOMA probes geometric regularity, FLU-π probes local π-electron

fluctuation, ELF-π probes real-space π-electron localization, and AV1245 probes cyclic multicenter π-electron delocalization. Their consistent nonaromatic character across independent physical criteria makes it unlikely that the conclusion is caused by the limitation of any single descriptor. At the same time, these thresholds are empirical guidelines rather than strict theoretical criteria, and no finite set of descriptors can provide a formal proof of the absence of aromaticity. Therefore, the present results are best described as providing reasonable converging evidence against aromaticity in the C_{12} ring within the limitations of the employed descriptors.

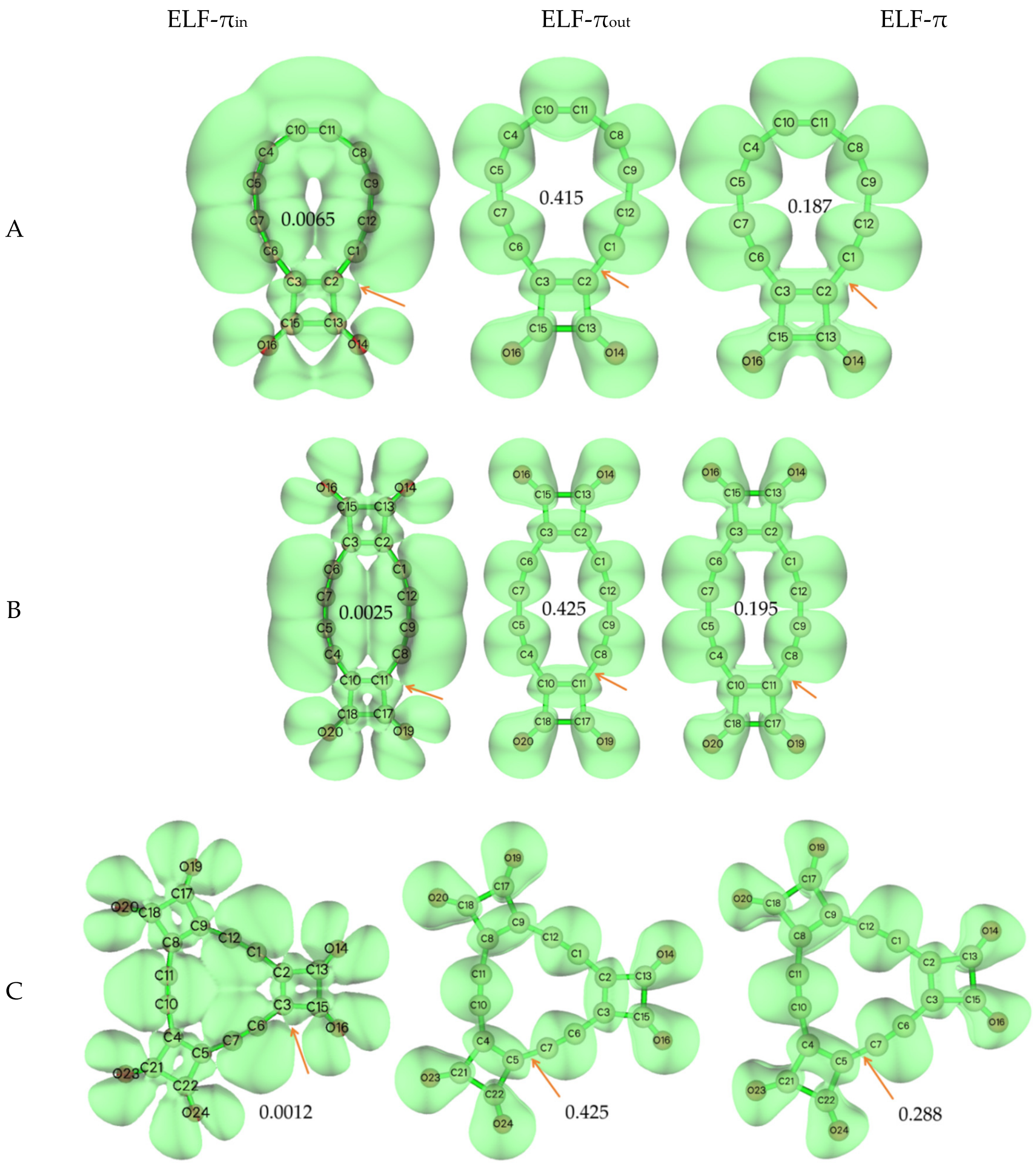

Figure S9. Isosurface contour of ELF-π_{in}, ELF-π_{out}, and ELF-π of C_{12}-$(CO)_n$ and their bifurcation points (A: n = 2; B: n = 4 ; C: n = 6). All values in a.u.

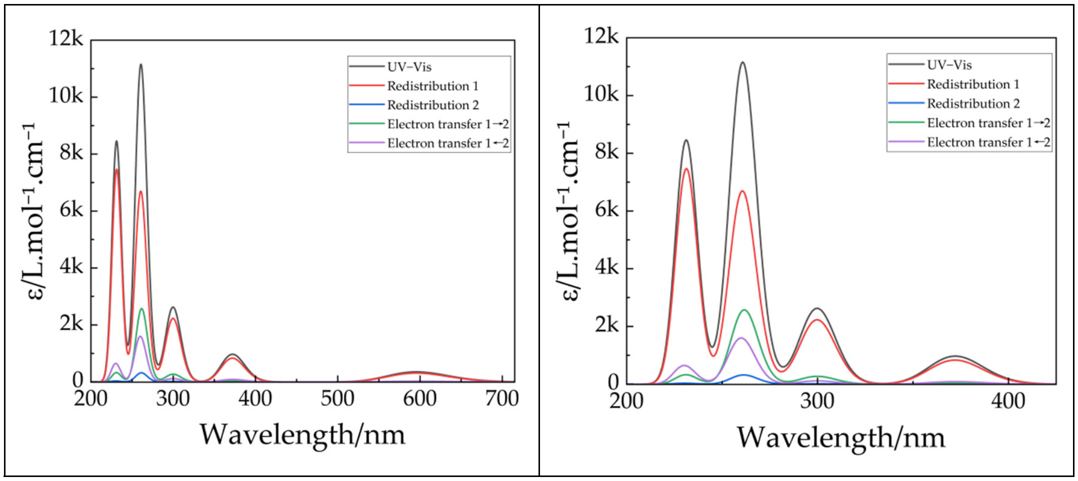

Figure S10. Simulated absorption spectra (black curves) and charge-transfer spectra (CTSs, colored curves) of C_{12}–$(CO)_2$ (200–715 nm, left; 200–425 nm, right). Gaussian function with a full width at half-maximum (FWHM) of 0.3 eV was employed for broadening the theoretically calculated data as a spectrum curve. Fragment 1 and 2 are C_{12} ring and $(CO)_2$, respectively.

Table S6 (a). Excited energy (in eV), excited wavelength (in nm), oscillator strength (*f*) and main molecular orbital transitions of 20 excited states of C_{12}-$(CO)_2$ at TD-ωB97XD/def2-TZVP level (based on the optimized structure at the level of ωB97XD/def2-TZVP)

State	Energy/eV	λ/nm	*f*	Mayor MO transitions
1	1.8245	679.55	0	H→L 94.7%
2	2.0820	595.51	0.0039	H-1→L 94.7%
3	2.9031	427.08	0	H-1→L+1 89.8%
4	3.0939	400.74	0	H-2→L 75.9%, H→L+3 15.5%
5	3.3089	374.70	0	H→L+2 53.2%, H-3→L 23.5%, H-2→L+2 17.1%
6	3.3332	371.97	0.0102	H→L+1 79.6%, H-2→L+1 6.8%
7	3.5951	344.87	0	H-3→L 64.5%, H-2→L+2 18.5%, H→L+2 7.9%
8	4.1343	299.89	0.0290	H-4→L 34.5%, H→L+4 22.8%, H-1→L+2 18.5%, H-3→L+1 15.5%
9	4.3191	287.06	0.0006	H-1→L+4 71.9%, H-4→L+1 21.8%
10	4.6885	264.44	0	H-7→L 29.2%, H→L+3 25.7%, H-1→L+5 15.5%, H-2→L+3 11.1%
11	4.7362	261.78	0.0710	H-1→L+2 62.3%, H-4→L 32.6%
12	4.7773	259.53	0.0547	H-5→L 35.0%, H-2→L+1 20.4%, H→L+5 11.9%, H→L+1 11.1%, H-1→L+3 8.5%
13	4.8324	256.57	0	H-1→L+5 51.9%, H→L+3 12.5%, H-5→L+1 10.9%, H-7→L 7.0%
14	5.0857	243.79	0.0001	H-2→L+2 41.2%, H→L+2 31.2%, H→L+6 12.2%, H-3→L 7.1%
15	5.2069	238.12	0	H→L+3 31.4%, H-7→L 18.4%, H-6→L+2 15.2%, H-2→L 11.3%, H-3→L+2 8.1%
16	5.3359	232.36	0.0597	H→L+4 45.0%, H-3→L+1 44.0%
17	5.3434	232.03	0.0001	H-6→L 80.5%, H-6→L+3 8.3%

18	5.3763	230.61	0.0123	H-5→L 41.8%, H-1→L+3 36.0%, H→L+5 14.6%
19	5.4225	228.65	0.0261	H→L+5 43.7%, H-1→L+3 14.8%, H-2→L+1 13.1%, H-2→L+5 12.3%
20	5.6725	218.57	0.0002	H-4→L+1 73.6%, H-1→L+4 22.3%

Table S6 (b). Excited energy (in eV), excited wavelength (in nm), oscillator strength (*f*) and main molecular orbital transitions of 20 excited states of C_{12}-$(CO)_4$ at TD-ωB97XD/def2-TZVP level (based on the optimized structure at the level of ωB97XD/def2-TZVP)

State	Energy/eV	λ/nm	*f*	Mayor MO transitions
1	2.0231	612.84	0	H→L 88.9%, H-2→L+2 6.1%
**2**	2.1278	582.69	0	H-1→L 97.1%
3	2.7864	444.96	0	H-2→L 72.1%, H→L+2 23.4%
**4**	2.8993	427.63	0	H-3→L 85.4%, H→L+1 9.0%
5	3.3732	367.56	0	H→L+1 55.4%, H-2→L+4 27.6%, H-3→L 8.4%
6	3.4937	354.88	0.0005	H-2→L+1 49.5%, H→L+4 39.7%
7	3.7560	330.10	0	H-4→L 68.2%, H-2→L+2 18.0%
8	4.0601	305.37	0.2100	H-5→L 44.1%, H-1→L+1 21.8%, H→L+5 14.6%, H-3→L+3 12.5%
9	4.1377	299.65	0	H-1→L+3 89.4%, H-5→L+5 6.8%
10	4.4597	278.01	0.0981	H-1→L+1 60.6%, H-5→L 35.5%
11	4.4718	277.26	0	H-1→L+5 84.3%, H-5→L+3 12.8%
12	4.7549	260.75	0	H→L+3 78.7%, H-3→L+5 7.2%, H-4→L+3 6.4%
13	4.7829	259.22	0.3075	H-1→L+2 50.6%, H-6→L 37.6%, H-2→L+3 6.2%
14	4.8124	257.63	0	H→L+2 63.0%, H-2→L 21.4%, H-4→L+2 7.9%
15	4.9801	248.96	0.0004	H-3→L+2 41.6%, H-7→L 26.0%, H-9→L 25.2%
16	5.1135	242.46	0.0001	H-6→L 52.9%, H-1→L+2 43.3%
17	5.2702	235.26	0	H-8→L 48.3%, H→L+1 13.5%, H-4→L+1 12.9%, H-7→L+2 11.6%, H-2→L+4 5.2%
18	5.3445	231.98	0	H-3→L+1 65.5%, H-4→L 11.7%, H-7→L+4 7.6%, H-8→L+1 6.3%
19	5.3856	230.21	0	H-7→L+1 23.8%, H-12→L 21.2%, H-4→L+2 17.7%, H-8→L+4 14.6%, H-2→L+6 8.1%
20	5.4271	228.45	0.0001	H-7→L 52.7%, H-8→L+2 15.0%, H-3→L+2 14.3%, H-9→L 13.5%

Table S6 (c). Excited energy (in eV), excited wavelength (in nm), oscillator strength (*f*) and main molecular orbital transitions of 20 excited states of C_{12}-$(CO)_6$ at TD-ωB97XD/def2-TZVP level (based on the optimized structure at the level of ωB97XD/def2-TZVP)

State	Energy/eV	λ/nm	*f*	Mayor MO transitions
1	1.9882	623.60	0	H→L 87.2%
**2**	2.1394	579.53	0	H-1→L 97.6%
3	2.5597	484.37	0	H-3→L 75.2%, H→L+2 12.5%
**4**	2.5597	484.37	0	H-2→L 75.2%, H→L+1 12.5%

5	3.3151	374.00	0	H→L+1 34.4%, H-3→L+3 14.7%, H-2→L+1 14.1%, H-3→L+2 14.1%, H→L+4 10.1%
6	3.3151	374.00	0	H→L+2 34.4%, H-2→L+3 14.7%, H-3→L+1 14.1%, H-2→L+2 14.1%, H→L+5 10.1%
7	3.4606	358.27	0.0005	H-2→L+2 27.8%, H-3→L+1 27.8%, H→L+3 24.0%, H-2→L+5 7.1%, H-3→L+4 7.1%
8	3.9340	315.16	0	H-7→L 64.3%, H-11→L 7.6%
9	3.9340	315.16	0	H-6→L 64.3%, H-10→L 7.6%
10	4.2723	290.20	0	H-9→L 61.8%, H-3→L+5 5.2%, H-2→L+4 5.2%, H-3→L+ 2 5.1%, H-2→L+1 5.1%, H-6→L+1 5.1%, H-7→L+2 5.1%
11	4.4168	280.71	0.7228	H-1→L+2 73.4%, H-5→L 13.8%
12	4.4168	280.71	0.7228	H-1→L+1 73.4%, H-4→L 13.8%
13	4.5914	270.04	0.0250	H-5→L 75.9%, H-1→L+2 18.1%
14	4.5914	270.04	0.0250	H-4→L 75.9%, H-1→L+1 18.1%
15	4.7360	261.79	0	H→L+1 26.3%, H-2→L 18.1%, H→L+4 16.8%, H-6→L 10.1%, H-3→L+2 7.3%, H-2→L+1 7.3%
16	4.7360	261.79	0	H→L+2 26.3%, H-3→L 18.1%, H→L+5 16.8%, H-7→L 10.1%, H-2→L+2 7.3%, H-3→L+1 7.3%
17	5.1066	242.79	0	H-2→L+1 25.9%, H-3→L+2 25.9%, H-9→L 14.4%, H→L 11.7%
18	5.1814	239.29	0.0004	H-8→L 77.6%
19	5.1934	238.73	0	H-10→L 72.8%
20	5.1934	238.73	0	H-11→L 72.8%

Table S7 (a). Excited energy (in eV), excited wavelength (in nm), oscillator strength (*f*) and main molecular orbital transitions of 20 excited states of C_{12}-$(CO)_2$ at TD-CAM-B3LYP/def2-TZVP level (based on the optimized structure at the ωB97XD/def2-TZVP level)

State	Energy/eV	λ/nm	*f*	Mayor MO transitions
1	1.8361	675.26	0	H→L 95.3%
2	2.0700	598.96	0.0040	H-1→L 94.8%
3	2.8674	432.39	0	H-1→L+1 91.7%
4	3.1095	398.73	0	H-2→L 79.2%, H→L+3 13.8%
5	3.3184	373.63	0.0101	H→L+1 81.4%, H-2→L+1 5.2%
6	3.3246	372.93	0.0006	H→L+2 51.4%, H-3→L 26.9%, H-2→L+2 15.6%
7	3.6034	344.08	0	H-3→L 61.3%, H-2→L+2 20.2%, H→L+2 9.5%
8	4.1137	301.39	0.0313	H-4→L 33.1%, H→L+4 23.2%, H-1→L+2 20.3%, H-3→L+1 15.2%
9	4.2766	289.91	0.0006	H-1→L+4 72.9%, H-4→L+1 21.1%
10	4.6669	265.67	0	H→L+3 32.9%, H-7→L 25.4%, H-1→L+5 14.6%, H-2→L+3 9.0%
11	4.7001	263.79	0.0754	H-1→L+2 62.6%, H-4→L 33.2%
12	4.7405	261.54	0.0476	H-5→L 34.9%, H-2→L+1 26.4%, H→L+1 10.5%, H→L+5 9.8%, H-1→L+3 7.3%

13	4.8089	257.82	0	H-1→L+5 52.0%, H→L+3 15.3%, H-5→L+1 11.9%
14	5.0250	246.73	0	H-2→L+2 43.4%, H→L+2 33.0%, H→L+6 9.6%, H-3→L 7.0%
15	5.1491	240.79	0	H→L+3 26.4%, H-7→L 25.1%, H-6→L+2 12.9%, H-2→L 9.2%, H-3→L+2 7.8%, H-2→L+3 7.4%
16	5.2662	235.43	0.0015	H-1→L+3 49.2%, H-5→L 41.0%, H-2→L+1 5.7%
17	5.2938	234.21	0.0569	H→L+4 46.2%, H-3→L+1 44.7%
18	5.3791	230.49	0.0001	H-6→L 81.4%, H-6→L+3 6.4%
19	5.4029	229.48	0.0137	H→L+5 59.6%, H-2→L+1 13.3%, H-2→L+5 11.5%
20	5.6241	220.45	0.0002	H-4→L+1 74.5%, H-1→L+4 21.9%

Table S7 (b). Excited energy (in eV), excited wavelength (in nm), oscillator strength (*f*) and main molecular orbital transitions of 20 excited states of C_{12}-$(CO)_4$ at TD-CAM-B3LYP/ def2-TZVP level (based on the optimized structure at the level of ωB97XD/def2-TZVP)

State	Energy/eV	λ/nm	*f*	Mayor MO transitions
1	2.0389	608.09	0	H-1→L 90.4%
**2**	2.1094	587.77	0	H→L 97.6%
3	2.8039	442.18	0	H-2→L 75.7%, H-1→L+2 20.1%
**4**	2.9027	427.13	0	H-3→L 86.2%, H-1→L+1 8.7%
5	3.3965	365.04	0	H-1→L+1 57.1%, H-2→L+4 26.0%, H-3→L 7.9%
6	3.5238	351.85	0.0005	H-2→L+1 50.0%, H-1→L+4 39.0%
7	3.7664	329.18	0	H-4→L 69.8%, H-2→L+2 16.2%
8	4.0407	306.84	0.2203	H-5→L 41.6%, H→L+1 24.6%, H-1→L+5 14.9%, H-3→L+3 12.4%
9	4.1054	302.00	0	H→L+3 89.4%, H-5→L+5 6.8%
10	4.4240	280.25	0	H→L+5 84.9%, H-5→L+3 12.5%
11	4.4427	279.07	0.0946	H→L+1 59.3%, H-5→L 37.9%
12	4.6591	266.11	0	H-1→L+2 69.4%, H-2→L 20.0%
13	4.7424	261.44	0	H-1→L+3 80.2%, H-3→L+5 7.0%, H-4→L+3 5.3%
14	4.7816	259.29	0.3041	H→L+2 58.3%, H-6→L 29.9%, H-2→L+3 6.8%
15	4.9542	250.26	0.0033	H-6→L 61.1%, H→L+2 35.8%
16	4.9974	248.10	0.0004	H-3→L+2 43.5%, H-7→L 26.4%, H-9→L 22.9%
17	5.2575	235.82	0	H-8→L 40.9%, H-1→L+1 17.3%, H-4→L+1 15.7%, H-2→L+4 9.1%, H-7→L+2 8.1%
18	5.2984	234.00	0	H-2→L+2 31.6%, H-4→L 19.6%, H-8→L+1 14.6%, H-7→L+4 10.4%, H-1→L+6 9.5%, H-1→L 5.2%
19	5.3466	231.89	0	H-3→L+1 81.4%, H-2→L+2 9.0%
20	5.4308	228.30	0.0001	H-7→L 56.2%, H-3→L+2 16.9%, H-8→L+2 11.4%, H-9→L 10.6%

Table S7 (c). Excited energy (in eV), excited wavelength (in nm), oscillator strength (*f*) and

main molecular orbital transitions of 20 excited states of C_{12}-$(CO)_6$ at TD-CAM-B3LYP/ def2-TZVP level (based on the optimized structure at the level of ωB97XD/def2-TZVP)

State	Energy/eV	λ/nm	f	Mayor MO transitions
1	2.0012	619.55	0	H→L 89.2%
**2**	2.1113	587.24	0	H-1→L 98.1%
3	2.5706	482.32	0	H-3→L 78.8%, H→L+1 10.8%
**4**	2.5706	482.32	0	H-2→L 78.8%, H→L+2 10.8%
5	3.3325	372.05	0	H→L+2 37.4%, H-3→L+3 13.5%, H-2→L+2 13.5%, H-3→L+1 13.5%, H→L+4 9.1%
6	3.3325	372.05	0	H→L+1 37.4%, H-2→L+3 13.5%, H-2→L+1 13.5%, H-3→L+2 13.5%, H→L+5 9.1%
7	3.4842	355.85	0.0005	H-3→L+2 28.4%, H-2→L+1 28.4%, H→L+3 22.8%, H-3→L+4 6.8%, H-2→L+5 6.8%
8	3.9539	313.57	0	H-7→L 65.8%, H-11→L 5.7%
9	3.9539	313.57	0	H-6→L 65.8%, H-10→L 5.7%
10	4.2947	288.69	0	H-8→L 60.7%, H-3→L+1 5.7%, H-2→L+2 5.7%, H-6→L+2 5.2%, H-7→L+1 5.2%, H-2→L+4 5.1%, H-3→L+5 5.1%
11	4.3868	282.63	0.6513	H-1→L+2 85.8%
12	4.3868	282.63	0.6512	H-1→L+1 85.8%
13	4.5295	273.73	0.1015	H-4→L 86.5%, H-1→L+2 6.8%
14	4.5295	273.73	0.1015	H-5→L 86.5%, H-1→L+1 6.8%
15	4.6001	269.53	0	H→L+1 27.2%, H→L+5 16.1%, H-3→L 15.8%, H-7→L 11.3%, H-2→L+1 9.1%, H-3→L+2 9.1%
16	4.6001	269.53	0	H→L+2 27.2%, H→L+4 16.1%, H-2→L 15.8%, H-6→L 11.3%, H-2→L+2 9.1%, H-3→L+1 9.1%
17	4.9432	250.82	0	H-2→L+2 28.4%, H-3→L+1 28.4%, H-8→L 16.3%, H→L 10.0%
18	5.1887	238.95	0.0004	H-9→L 81.8%
19	5.2035	238.27	0	H-10→L 77.7%
20	5.2035	238.27	0	H-11→L 77.7%

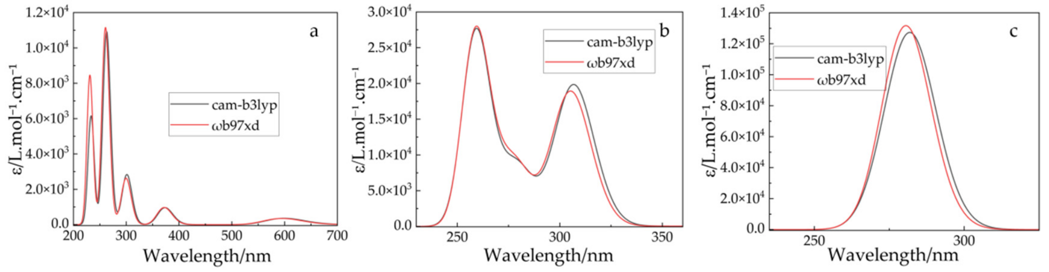

Figure S11. Simulated UV–Vis spectra of C_{12}-$(CO)_n$ (n = 2 (a), n = 4 (b), and n = 6 (c)) with FWHM 0.3 eV (The UV–Vis spectra originate from TD-CAM-B3LYP/def2-TZVP and TD-ωB97XD/def2-TZVP levels based on the same structure optimized at the ωB97XD/ def2-TZVP level.).

Table S8. Excited energy, excited wavelength, oscillator strength (f) and main molecular

orbital transitions of low-lying excited states of C_{12}-$(CO)_n$ (n = 2, 4, and 6) obtained from TD-CAM-B3LYP/def2-TZVP and TD-ωB97XD/def2-TZVP levels.

Compound	Functional	State	Energy /eV	λ/nm	*f*	Mayor MO transitions
C_{12}–$(CO)_n$	TD-ωB97XD	2	2.0820	595.51	0.0039	H-1→L 94.7%
	TD-CAM-B3LYP	2	2.0700	598.96	0.0040	H-1→L 94.8%
C_{12}–$(CO)_n$	TD-ωB97XD	8	4.0601	305.37	0.2100	H-5→L 44.1%, H-1→L+1 21.8%, H→L+5 14.6%, H-3→L+3 12.5%
	TD-CAM-B3LYP	8	4.0407	306.84	0.2203	H-5→L 41.6%, H→L+1 24.6%, H-1→L+5 14.9%, H-3→L+3 12.4%
C_{12}–$(CO)_n$	TD-ωB97XD	11	4.4168	280.71	0.7228	H-1→L+2 73.4%, H-5→L 13.8%
		12	4.4168	280.71	0.7228	H-1→L+1 73.4%, H-4→L 13.8%
	TD-CAM-B3LYP	11	4.3868	282.63	0.6513	H-1→L+2 85.8%
		12	4.3868	282.63	0.6512	H-1→L+1 85.8%

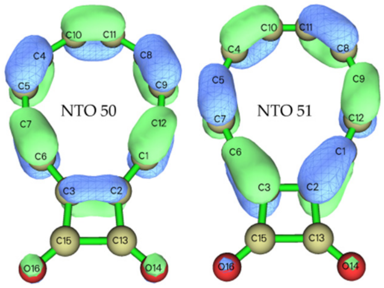

Figure S12(a). Natural transition orbital (NTO) pair for S0→S2 for C_{12}-$(CO)_2$ with contribution rate 95.79% (based on the calculation results at the TD-ωB97XD/def2-TZVP level). Isovalue = 0.05 a.u. Green and blue regions denote positive and negative orbital phases, respectively.

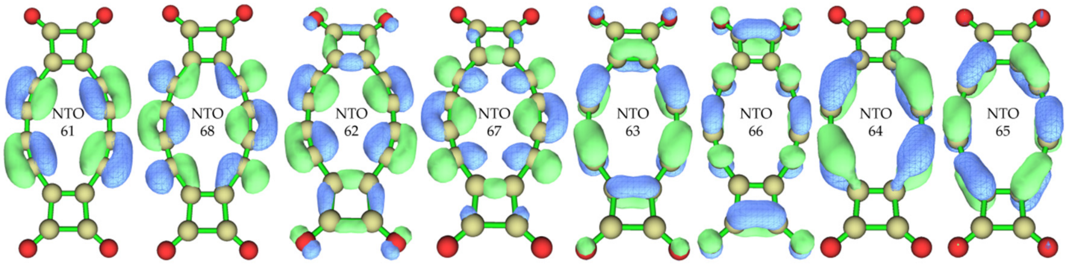

Figure S12(b). Natural transition orbital (NTO) pair for S0→S8 for C_{12}-$(CO)_4$ based on the calculation results at the TD-ωB97XD/def2-TZVP level. The contributions of NTO 61→NTO 68, NTO 62→NTO 67, NTO 63→NTO 66, and NTO 64→NTO 65 are 12.61%, 17.83%, 22.67%, and 44.80%, respectively. Isovalue = 0.05 a.u. Green and blue regions denote positive and negative orbital phases, respectively.

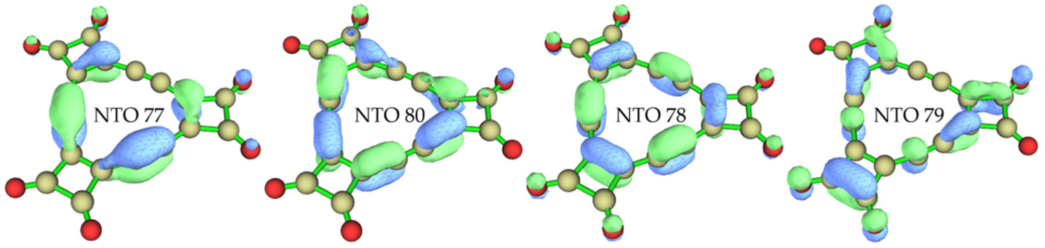

Figure S12(c). Natural transition orbital (NTO) pair for S0→S11 for C_{12}-$(CO)_6$ based on the calculation results at the TD-ωB97XD/def2-TZVP level. The contributions of NTO 77→NTO 80 and NTO 78→NTO 79 are 15.46% and 74.18%, respectively. Isovalue = 0.05 a.u. Green and blue regions denote positive and negative orbital phases, respectively.

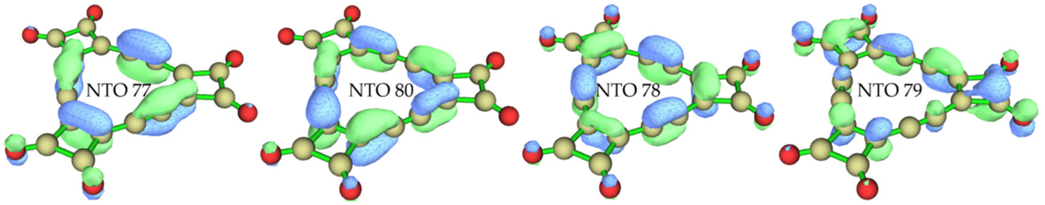

Figure S12(d). Natural transition orbital (NTO) pair for S0→S12 for C_{12}-$(CO)_6$ based on the calculation results at the TD-ωB97XD/def2-TZVP level. The contributions of NTO 77→NTO 80 and NTO 78→NTO 79 are 14.84% and 74.29%, respectively. Isovalue = 0.05 a.u. Green and blue regions denote positive and negative orbital phases, respectively.

n	state	C_{ele} & C_{hole}	hole & electron	Sr
2	S2	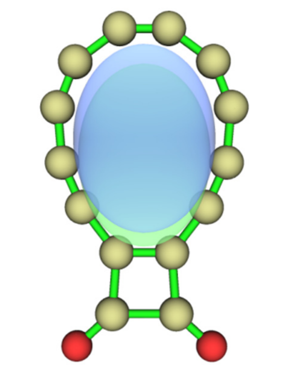	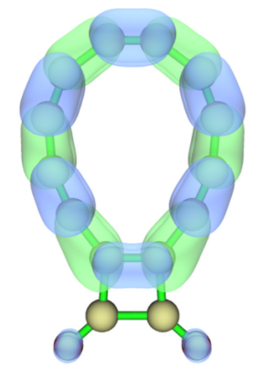	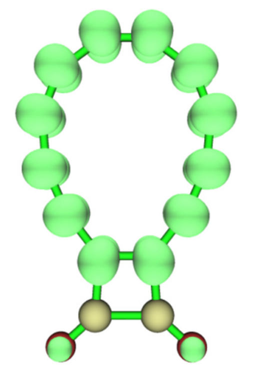

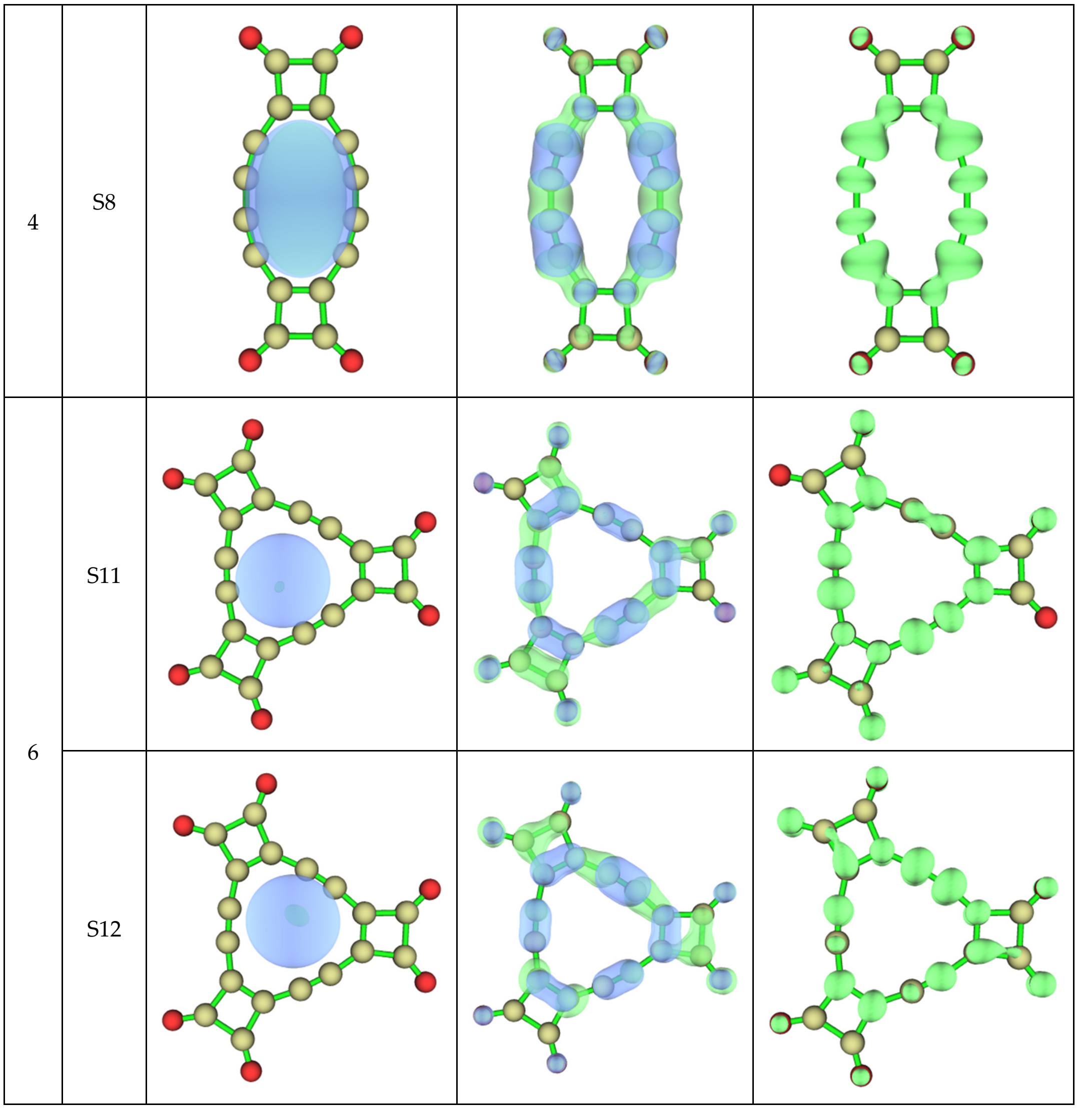

Figure S13. Excited-state hole-electron analyses for C_{12}-$(CO)_n$ (n = 2, 4, and 6) calculated at TD-ωB97XD/def2-TZVP level (based on the optimized structure at the ωB97XD/def2-TZVP level). C_{ele} & C_{hole} denote the isosurface plots showing smoothed representations of the electron and hole distributions. Hole & electron are the isosurface plots of the hole and electron distributions. Green and blue stand for electron and hole distributions, respectively. Isovalue = 0.002 a.u.

Text S2. Hole-electron analysis and assignment of locally excited states [19]

Hole-electron analysis was used to characterize the excited states. The hole distribution represents where the excited electron comes from, and the electron distribution represents where it goes to. Both distributions integrate to unity over all space and are positive everywhere. The descriptors are defined as follows:

Sr: integral of the Sr function, measuring the spatial overlap between the hole and electron distributions. Its value ranges from 0 to 1. Sr = 1 means perfect overlap, while Sr = 0 means no overlap.

D**:** distance between the centroids of the hole and electron distributions.

H**:** overall average spatial extent of the hole and electron distributions.

t**:** degree of hole–electron separation along the charge-transfer (CT) direction. t > 0 indicates significant separation, because the centroid distance is relatively large compared with the average spatial extent. t < 0 indicates no significant separation, because the centroid distance is not large relative to the average extent.

HDI/EDI: hole delocalization index and electron delocalization index, respectively. Smaller values indicate a higher degree of delocalization, i.e., a more uniform distribution.

E_{coul}: Coulomb attractive energy between the hole and electron.

A locally excited (LE) state is typically characterized by a small D, a relatively large Sr (> 0.5), a negative t, comparable HDI and EDI values, and strong spatial overlap of the hole and electron distributions without obvious separation into distinct donor and acceptor regions. In contrast, a CT state generally shows a large D, a small Sr, a positive t, and spatially separated hole and electron distributions.

For the excited states discussed in this work, the calculated descriptors in **Table 3** satisfy the LE criteria: all have small D, large Sr (about 0.8), and negative t. The HDI and EDI values are close to each other, and the hole and electron distributions in **Figure S10** overlap strongly over the C_{12} ring and the –$(CO)_n$ group. These states are therefore assigned as LE states.

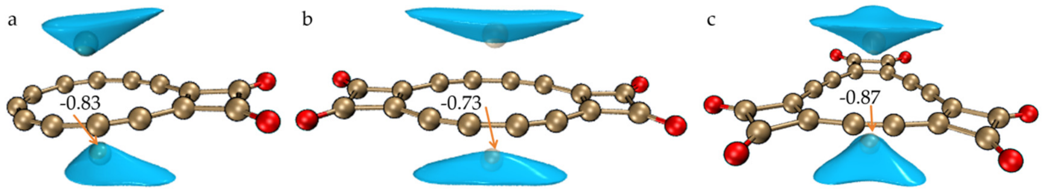

Figure S14(a). Isosurface of the vdW potential of C_{12}-$(CO)_2$ (a), C_{12}-$(CO)_4$ (b), and C_{12}-$(CO)_6$ (c), with He as the probe atom (isovalue = –0.5 kcal·mol^{-1}). Light brown spheres: C; red spheres: O. Small spheres inside blue isosurfaces denote vdW potential minima. Orange arrows point to these minima.

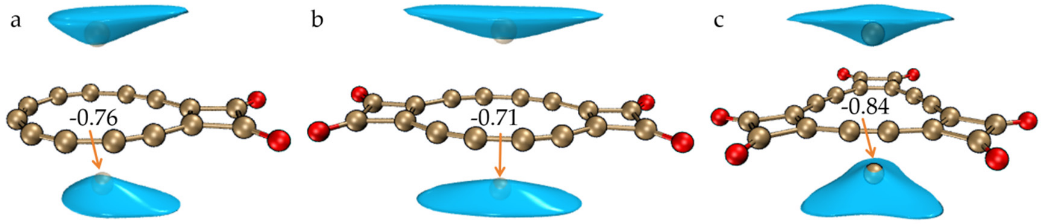

Figure S14(b). Isosurface of the vdW potential of C_{12}-$(CO)_2$ (a), C_{12}-$(CO)_4$ (b), and C_{12}-$(CO)_6$ (c),

with Ne as the probe atom (isovalue = –0.5 kcal·mol^{-1}). Light brown spheres: C; red spheres: O. Small spheres inside blue isosurfaces denote vdW potential minima. Orange arrows point to these minima.

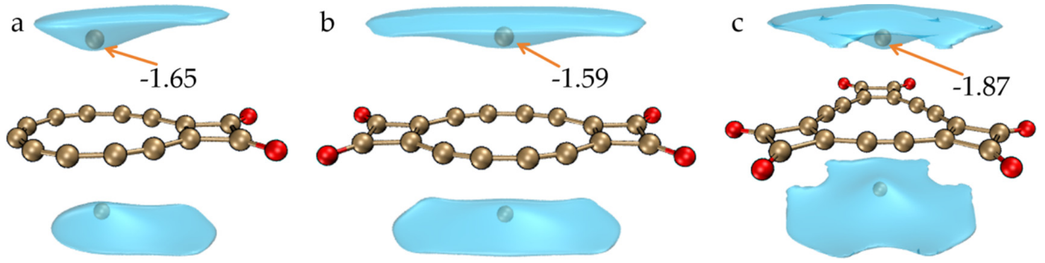

Figure S14(c). Isosurface of the vdW potential of C_{12}-$(CO)_2$ (a), C_{12}-$(CO)_4$ (b), and C_{12}-$(CO)_6$ (c), with Ar as the probe atom (isovalue = –1.0 kcal·mol^{-1}). Light brown spheres: C; red spheres: O. Small spheres inside blue isosurfaces denote vdW potential minima. Orange arrows point to these minima.

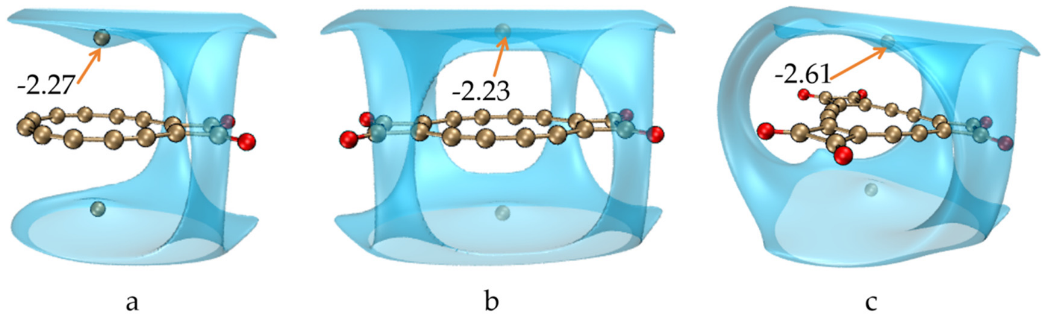

Figure S14(d). Isosurface of the vdW potential of C_{12}-$(CO)_2$ (a), C_{12}-$(CO)_4$ (b), and C_{12}-$(CO)_6$ (c), with Xe as the probe atom (isovalue = –1.0 kcal·mol^{-1}). Light brown spheres: C; red spheres: O. Small spheres inside blue isosurfaces denote vdW potential minima. Orange arrows point to these minima.

Table S9. The van der Waals potential minima (VvdW, in kcal·mol^{-1}) and vertical distances between noble-gas atom and molecular plane(deq, in Å) for noble-gas probes over C_{12}-$(CO)_n$ substrates.

probe	C_{12}-$(CO)_2$	C_{12}-$(CO)_4$	C_{12}-$(CO)_6$
He	−0.83 (1.91)	−0.73 (2.19)	−0.87 (1.93)
Ne	−0.76 (2.62)	−0.71 (2.81)	−0.84 (2.63)
Ar	−1.65 (3.02)	−1.59 (3.17)	−1.87 (3.04)
Xe	−2.27 (3.32)	−2.23 (3.49)	−2.61 (3.28)

Table S10. SAPT0 energy decomposition results for the C_{12}-$(CO)_n$@He/Ne complex at the SAPT0/jun-cc-pVDZ level. All energies are given in kcal·mol^{-1}.

	E_{elst}	E_{exch}	E_{ind}	E_{disp}	E_{SAPT0}	E_{sSAPT0}
C_{12}-$(CO)_2$@He	−0.03974	0.18420	−0.01181	−0.17984	−0.04719	−0.04718
C_{12}-$(CO)_4$@He	−0.04354	0.17613	−0.01279	−0.16718	−0.04738	−0.04737
C_{12}-$(CO)_6$@He	−0.05355	0.21830	−0.02002	−0.18691	−0.04218	−0.04216

C_{12}-$(CO)_2$@Ne	−0.13368	0.43680	−0.01277	−0.35639	−0.06604	−0.06603
C_{12}-$(CO)_4$@Ne	−0.14297	0.46717	−0.01600	−0.34460	−0.03639	−0.03635
C_{12}-$(CO)_6$@Ne	−0.18271	0.54374	−0.02404	−0.37854	−0.04155	−0.04141

Note: E_{elst}, E_{exch}, E_{ind}, and E_{disp} represent electrostatic energy, exchange repulsion energy, induction energy, and dispersion energy, respectively.

Table S11. Raw interaction energies ΔE_{raw}, counterpoise corrected interaction energies ΔE_{CP}, and BSSE values (unit: kcal·mol^{-1}) for C_{12}-$(CO)_n$@He/Ne (n = 2,4,6) calculated using different methods.

Method		ΔE_{raw}	ΔE_{CP}	BSSE		ΔE_{raw}	ΔE_{CP}	BSSE
ωB97X-D3BJ/def2-TZVP	2@He	–0.543	–0.392	0.151	2@Ne	–1.116	–0.739	0.377
ωB97X-D3BJ/def2-QZVP		–0.405	–0.393	0.012		–0.800	–0.748	0.052
ωB97X-D3BJ/ma-def2-TZV		–0.410	–0.404	0.006		–	–	–
CAM-B3LYP-D3BJ/def2-TZ		–0.495	–0.341	0.154		–1.105	–0.731	0.374
ωB97X-D3BJ/def2-TZVP	4@He	–0.488	–0.350	0.138	4@Ne	–1.044	–0.675	0.369
ωB97X-D3BJ/def2-QZVP		–0.359	–0.346	0.0125		–0.728	–0.671	0.057
CAM-B3LYP-D3BJ/def2-TZ		–0.443	–0.302	0.141		–1.052	–0.686	0.367
ωB97X-D3BJ/def2-TZVP	6@He	–0.476	–0.328	0.148	6@Ne	–1.113	–0.713	0.401
ωB97X-D3BJ/def2-QZVP		–0.336	–0.323	0.013		–0.764	–0.706	0.058
CAM-B3LYP-D3BJ/def2-TZ		–0.443	–0.293	0.150		–1.117	–0.719	0.398

Note: For brevity, 2@He denotes C_{12}-$(CO)_2$@He, and other notation follows the same convention.

Table S12. Vertical distances (Å) between the noble-gas atom and the C_{12} ring for C_{12}-$(CO)_n$@He/Ne (n=2,4,6).

Complex	2@He	2@Ne	4@He	4@Ne	6@He	6@Ne
Distance1	2.886	2.889	3.063	3.026	3.012	2.949
Distance2	1.91	2.62	2.19	2.81	1.93	2.63

Note: Distance1 and Distance2 denote the vertical distances from the noble gas atom to the C_{12} ring. Distance1 corresponds to the value obtained from geometry optimization at the ωB97XD/def2-TZVP level. Distance2 represents the result calculated from van der Waals potential using the UFF force field. For brevity, 2@He denotes C_{12}-$(CO)_2$@He, and other notation follows the same convention.

Table S13. Optimized Cartesian coordinates for C_{12}-$(CO)_n$@He/Ne (n = 2,4,6) at the ωB97XD/ def2-TZVP level. The values are given in Å.

C_{12}-$(CO)_2$@He (charge = 0, spin multiplicity = 1)			
Atom	x	y	z
C	0.05441800	0.42295700	1.53635300
C	0.03958100	1.54196600	0.68272000
C	0.03958100	1.54196600	-0.68272000
C	0.09319400	-3.17270700	-1.70763900

C	0.08246900	-2.04272200	-2.14702700
C	0.05441800	0.42295700	-1.53635300
C	0.06774900	-0.67927000	-2.03202700
C	0.09319400	-3.17270700	1.70763900
C	0.08246900	-2.04272200	2.14702700
C	0.10071700	-3.98894500	-0.60721600
C	0.10071700	-3.98894500	0.60721600
C	0.06774900	-0.67927000	2.03202700
C	0.01876000	3.05150800	0.78651300
O	0.00764300	3.84615500	1.66176700
C	0.01876000	3.05150800	-0.78651300
O	0.00764300	3.84615500	-1.66176700
He	-2.80247600	-1.56595900	0.00000000

C_{12}-$(CO)_4$@He (charge = 0, spin multiplicity = 1)			
Atom	x	y	z
C	1.47769500	-0.05030400	1.84775200
C	0.68316000	-0.04782300	3.00573200
C	-0.68312700	-0.04786800	3.00572500
C	-1.47768300	-0.05038600	-1.84776300
C	-1.79633300	-0.05247500	-0.68316500
C	-1.47768300	-0.05038600	1.84776300
C	-1.79633300	-0.05247500	0.68316500
C	1.47769500	-0.05030400	-1.84775200
C	1.79638800	-0.05255500	-0.68316800
C	-0.68312700	-0.04786800	-3.00572500
C	0.68316000	-0.04782300	-3.00573200
C	1.79638800	-0.05255500	0.68316800
C	0.78900800	-0.04413500	4.51662800
O	1.66202500	-0.04206500	5.31160800
C	-0.78900100	-0.04418600	4.51661300
O	-1.66202800	-0.04218600	5.31158300
C	0.78900800	-0.04413500	-4.51662800
C	-0.78900100	-0.04418600	-4.51661300
O	1.66202500	-0.04206500	-5.31160800
O	-1.66202800	-0.04218600	-5.31158300
He	-0.00061800	3.01239600	0.00000000

C_{12}-$(CO)_6$@He (charge = 0, spin multiplicity = 1)			
Atom	x	y	z
C	-1.55136300	1.59079600	-0.04191400
C	-0.68094200	2.69002200	-0.03978900
C	0.68094200	2.69002200	-0.03978900

C	1.98915600	-1.93472400	-0.03978900
C	2.67009800	-0.75529800	-0.03978900
C	1.55136300	1.59079600	-0.04191400
C	2.15335100	0.54812200	-0.04191400
C	-1.98915600	-1.93472400	-0.03978900
C	-2.67009800	-0.75529800	-0.03978900
C	0.60198800	-2.13891800	-0.04191400
C	-0.60198800	-2.13891800	-0.04191400
C	-2.15335100	0.54812200	-0.04191400
C	-0.78844100	4.20572900	-0.03661600
O	-1.66381200	4.99624700	-0.03504400
C	0.78844100	4.20572900	-0.03661600
O	1.66381200	4.99624700	-0.03504400
C	-4.03648900	-1.42005500	-0.03661600
C	-3.24804800	-2.78567400	-0.03661600
O	-5.15878300	-1.05722000	-0.03504400
O	-3.49497100	-3.93902700	-0.03504400
C	3.24804800	-2.78567400	-0.03661600
C	4.03648900	-1.42005500	-0.03661600
O	3.49497100	-3.93902700	-0.03504400
O	5.15878300	-1.05722000	-0.03504400
He	0.00000000	0.00000000	2.97079400

C_{12}-$(CO)_2$@Ne (charge = 0, spin multiplicity = 1)			
Atom	x	y	z
C	0.24225800	0.53637200	1.53677500
C	0.17753700	1.65326700	0.68271200
C	0.17753700	1.65326700	-0.68271200
C	0.42603800	-3.05417700	-1.70817200
C	0.37119000	-1.92569000	-2.14811300
C	0.24225800	0.53637200	-1.53677500
C	0.30158200	-0.56390600	-2.03332500
C	0.42603800	-3.05417700	1.70817200
C	0.37119000	-1.92569000	2.14811300
C	0.46481700	-3.86874400	-0.60719100
C	0.46481700	-3.86874400	0.60719100
C	0.30158200	-0.56390600	2.03332500
C	0.08727100	3.16024200	0.78645700
O	0.03905800	3.95356500	1.66167700
C	0.08727100	3.16024200	-0.78645700
O	0.03905800	3.95356500	-1.66167700
Ne	-2.54732400	-1.45054200	0.00000000

C_{12}-$(CO)_4$@Ne (charge = 0, spin multiplicity = 1)			
Atom	x	y	z
C	1.84756900	1.47745400	-0.22742600
C	3.00559600	0.68319500	-0.22119300
C	3.00559600	-0.68319500	-0.22119300
C	-1.84756900	-1.47745400	-0.22742600
C	-0.68311000	-1.79637900	-0.23198600
C	1.84756900	-1.47745400	-0.22742600
C	0.68311000	-1.79637900	-0.23198600
C	-1.84756900	1.47745400	-0.22742600
C	-0.68311000	1.79637900	-0.23198600
C	-3.00559600	-0.68319500	-0.22119300
C	-3.00559600	0.68319500	-0.22119300
C	0.68311000	1.79637900	-0.23198600
C	4.51641600	0.78893800	-0.21124200
O	5.31148500	1.66186800	-0.20581100
C	4.51641600	-0.78893800	-0.21124200
O	5.31148500	-1.66186800	-0.20581100
C	-4.51641600	0.78893800	-0.21124200
C	-4.51641600	-0.78893800	-0.21124200
O	-5.31148500	1.66186800	-0.20581100
O	-5.31148500	-1.66186800	-0.20581100
Ne	0.00000000	0.00000000	2.79902800

C_{12}-$(CO)_6$@Ne (charge = 0, spin multiplicity = 1)			
Atom	x	y	z
C	-1.55121800	1.59061000	-0.18575600
C	-0.68094100	2.68989700	-0.18168000
C	0.68094100	2.68989700	-0.18168000
C	1.98904900	-1.93466100	-0.18168000
C	2.66999000	-0.75523700	-0.18168000
C	1.55121800	1.59061000	-0.18575600
C	2.15311700	0.54808900	-0.18575600
C	-1.98904900	-1.93466100	-0.18168000
C	-2.66999000	-0.75523700	-0.18168000
C	0.60190000	-2.13869900	-0.18575600
C	-0.60190000	-2.13869900	-0.18575600
C	-2.15311700	0.54808900	-0.18575600
C	-0.78838100	4.20548400	-0.17415500
O	-1.66360600	4.99618800	-0.16994900
C	0.78838100	4.20548400	-0.17415500
O	1.66360600	4.99618800	-0.16994900
C	-4.03624600	-1.41998400	-0.17415500
C	-3.24786500	-2.78550000	-0.17415500

O	-5.15862900	-1.05736900	-0.16994900
O	-3.49502300	-3.93882000	-0.16994900
C	3.24786500	-2.78550000	-0.17415500
C	4.03624600	-1.41998400	-0.17415500
O	3.49502300	-3.93882000	-0.16994900
O	5.15862900	-1.05736900	-0.16994900
Ne	0.00000000	0.00000000	2.76548200

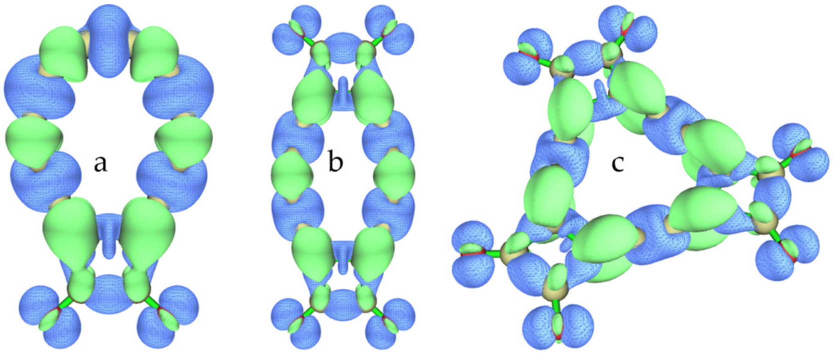

Figure S15. Isosurface maps of the orbital-weighted dual descriptor (OWDD) for C_{12}-$(CO)_n$: (a) (n = 2); (b) (n = 4); (c) (n = 6).. Green and blue denote regions with positive and negative values, respectively. Isovalue = 0.001 a.u.

Text S3. Additional information on LEAE and OWDD

As a rigorous energy-based descriptor, LEAE evaluates the intrinsic electron-accepting affinity of molecular surface sites; a more negative LEAE value indicates a greater tendency to accommodate external electrons and higher susceptibility toward nucleophilic attack. In addition, OWDD quantifies the relative electron-gain and electron-loss response of each site within the conceptual density functional theory (CDFT) framework. Positive OWDD values correspond to dominant electron-accepting behavior (nucleophilic-attack preference), whereas negative OWDD values reflect prevailing electron-donating character (electrophilic-attack preference). The dual-descriptor strategy eliminates the ambiguity of single-criterion evaluation and enables reliable discrimination of reactive site types.
